# Selective Interfacial Barriers Drive High‐Performance GeTe Thermoelectrics

**DOI:** 10.1002/advs.75403

**Published:** 2026-04-20

**Authors:** Liang‐Cao Yin, Xinhua Lu, Wei‐Di Liu, Meng Li, Siqi Liu, De‐Zhuang Wang, Hao Wu, Yuan‐Meng Liu, Xiao‐Lei Shi, Yifeng Wang, Lixiong Zhang, Qingfeng Liu, Zhi‐Gang Chen

**Affiliations:** ^1^ State Key Laboratory of Materials‐Oriented Chemical Engineering College of Chemical Engineering Nanjing Tech University Nanjing China; ^2^ School of Chemistry and Physics ARC Research Hub in Zero‐emission Power Generation for Carbon Neutrality, and Center for Materials Science Queensland University of Technology Brisbane Queensland Australia; ^3^ College of Materials Science and Engineering Nanjing Tech University Nanjing China

**Keywords:** carrier‐phonon decoupling, figure‐of‐merit, GeTe, thermoelectric

## Abstract

Thermoelectric technology provides a sustainable solution for harvesting waste heat. High‐efficiency waste heat recovery demands high figure of merit (*ZT*) materials, which are limited by intrinsic carrier and phonon coupling. Here, we introduce VSe_2_ nanowire precipitates to construct selective interfacial barriers for effective decoupling. Specifically, the electron transport is optimized by the synergistic effects of the minimization of the Fermi level mismatch between the VSe_2_ and GeTe, and a substantial reduction of interface state density via semi‐coherent interfaces. Concurrently, the significant phonon frequency mismatch at interfacial barriers effectively suppresses phonon transport. This approach yields a high *ZT* of 2.7 at 773 K, and record‐high average *ZT* of 1.9 across 300–773 K in the (Ge_0.82_Mn_0.04_Bi_0.04_Pb_0.1_Te)_0.99_(VSe_2_)_0.01_. The corresponding π‐type module exhibits a conversion efficiency of 11.4% under a 440 K temperature difference. This work highlights the potential of selective interfacial barriers for advancing thermoelectrics and energy‐harvesting applications.

## Graphical abstract

Carrier‐phonon decoupling is the key to developing high‐performance thermoelectric materials. Here, we demonstrate that selective interfacial barriers can decouple carrier and phonon transport, thus achieving an exceptional peak *ZT* of ∼2.7 at 773 K, along with a record‐high average *ZT* of 1.9 across 300–773 K in the (Ge_0.82_Mn_0.04_Bi_0.04_Pb_0.1_Te)_0.99_(VSe_2_)_0.01_.

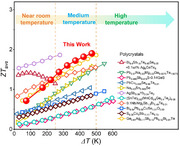

## 1 Introduction

The thermoelectric technology efficiently converts waste heat from industrial processes and vehicle exhaust into clean electricity through its solid‐state conversion mechanism without moving parts [1, 2]. This zero‐carbon emission energy conversion technology, with exceptionally long service life and near‐maintenance‐free operation, enables stable and continuous waste heat recovery and utilization, and provides a sustainable technological pathway to address global energy challenges [1, 2]. The conversion efficiency (*η*) of materials is directly linked to the dimensionless figure‐of‐merit (*ZT*), defined as *ZT = S^2^σT/κ*, incorporating the Seebeck coefficient (*S*), electrical conductivity (*σ*), thermal conductivity (*κ*), and the absolute temperature (*T*) [3]. The *κ* comprises two components: electronic thermal conductivity (*κ*_e_) and lattice thermal conductivity (*κ*_l_) [4]. The complex interdependence of key parameters, including the *S*, *σ*, *κ*, carrier concentration (*n*_h_), mobility (*µ*), and effective mass (*m* ^*^), fundamentally constrains the *ZT*, thereby refraining the *η* of sustainable waste heat recovery systems [5]. Traditional strategies typically focus on optimizing only individual parameters, such as fine‐tuning *n*_h_ or enhancing phonon scattering through nanoprecipitates [6]. In recent decades, efforts have increasingly shifted toward decoupling these interrelated properties to achieve further improvements in *ZT* [5, 7, 8, 9]. For example, from an electrical performance perspective, band convergency enhances *m* ^*^ by increasing valley degeneracy, which improves *S* and *S^2^σ* without significantly degrading *µ* [9]. Similarly, strategies that target both thermal and electrical performance focus on decoupling carrier‐phonon scattering and enhancing the ratio of weighted mobility *µ*_w_ to the *κ*_l_ (*µ*_w_/*κ*_l_), a critical objective in the development of advanced thermoelectric materials [7, 8, 10].

The most common strategy for decoupling carrier‐phonon scattering involves designing defects with an appropriate size, which deviates from the carrier mean free path (MFP) while aligning closely with the phonon MFP [11, 12, 13, 14]. For instance, in PbQ‐based (Q = Te, Se) materials, introducing the sub‐nanometer defects, such as interstitial atoms or an interstitial atom cluster, can effectively balance the trade‐off between *µ*_w_ and *κ*_l_. This approach leverages the smaller phonon MFP (0.1–10 nm) compared to the significantly larger carrier MFP (100–1000 nm) [12, 14]. However, such a strategy faces limitations in materials where the carrier and phonon MFPs are comparable. The optimization of electronic band or phonon structure separately (such as band convergence or phonon softening) has b ·een considered as an effective strategy for achieving carrier‐phonon decoupling, originating from the differences in their mechanisms within reciprocal space [15, 16]. For instance, the introduction of rare‐earth elements in GeTe can induce strongly correlated d, f electrons to achieve band convergence, while heavy atom (Pb, Bi) doping causes phonon softening, significantly optimizing *S^2^σ* and reducing *κ*_l_, thereby realizing carrier‐phonon decoupling [16]. However, the optimization of electronic band or phonon structure induced by multi‐component doping can easily introduce additional defects, which reduce the degree of carrier‐phonon decoupling and ultimately limit the thermoelectric performance of materials. Consequently, the key to achieving effective carrier‐phonon decoupling and superior thermoelectric performance lies in selectively blocking phonons while maintaining electron transport within a single structure.

In this study, we demonstrate that the selective interfacial barriers constructed solely by introducing VSe_2_ nanowire precipitates in GeTe can realize effective carrier‐phonon decoupling to achieve ultra‐high *ZT* and average *ZT* (*ZT*_ave_). This design effectively reduces the Fermi level (*E*_F_) mismatch at VSe_2_/GeTe interfaces by the similar *E*_F_ of VSe_2_ nanowire precipitates and GeTe, while utilizing semi‐coherent interfaces to significantly diminish interfacial dangling bonds, dislocation, and strain fields. This coordinated optimization of band and crystal structures significantly reduces potential fluctuations at heterointerfaces, thus effectively suppressing the formation of interfacial electron barriers and almost eliminating carrier scattering at the VSe_2_/GeTe interfaces. Simultaneously, these interfaces can establish an effective phonon scattering by phonon frequency mismatch, achieving carrier‐phonon decoupling. These strategies collectively lead to a high *Z* *T* of 2.7 at 773 K and the record‐high *ZT*_ave_ of ∼1.9 from 300 to 773 K in the (Ge_0.82_Mn_0.04_Bi_0.04_Pb_0.1_Te)_0.99_(VSe_2_)_0.01_. The corresponding *π*‐type module presents an outstanding *η* of ∼11.4% at ∆*T* of 440 K, which can directly harvest heat for sustainable power generation.

## 2 Result and Discussion

### 2.1 Selective Interfacial Barriers for High Thermoelectric Performance

**FIGURE 1:**
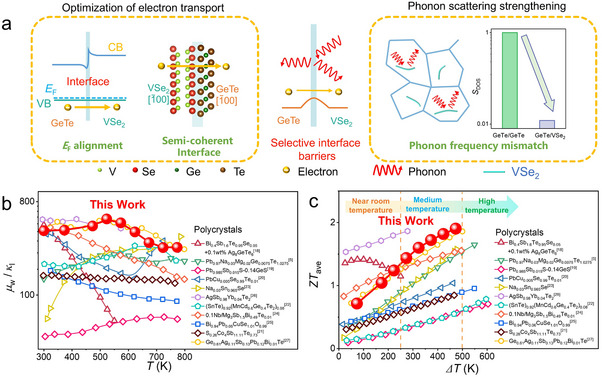
Selective interfacial barriers for high thermoelectric performance. (a) Schematic diagram of selective interfacial barriers, which can effectively enhance the electron transport and strengthen the phonon scattering. (b) Temperature‐dependent the ratio of weighted mobility to the lattice thermal conductive (*µ*_w_/*κ*_l_) and (c) Average figure‐of‐merit (*ZT*_ave_) of the (Ge_0.82_Mn_0.04_Bi_0.04_Pb_0.1_Te)_0.99_(VSe_2_)_0.01_, compared to other typical polycrystalline thermoelectric materials with high *ZT* [5, 18, 19, 20, 21, 22, 23, 24, 25, 26, 27] and high *ZT*_ave_ [5, 18, 19, 20, 21, 22, 23, 24, 25, 26, 27]. It should be noted that *ΔT* is defined as the difference between the measuring temperature and 300 K, and *ZT*_ave_ is calculated over the range from 300 K to the measuring temperature.

To form selective interfacial barriers in GeTe, we introduced VSe_2_ nanowire precipitates as the secondary phase, incorporated into our previously studied Ge_0.82_Mn_0.04_Bi_0.04_Pb_0.1_Te matrix [17] to effectively decouple carrier and phonon transport. This design utilizes VSe_2_ nanowires as multifunctional interfaces to synergistically modulate both band structure and crystal structure at heterointerfaces, effective maintaining electron transport and enhancing phonon scattering, thus achieving effectively carrier‐phonon decoupling. Specifically, such a design effectively reduces *E*_F_ mismatch through the similar *E*_F_ of VSe_2_ nanowire precipitates and GeTe matrix, while significantly decreasing dangling bond density, dislocations, and strain fields via semi‐coherent interfaces, thereby substantially diminishing interface potential fluctuations and enhancing electron transport. Simultaneously, an effective phonon scattering is caused by phonon frequency mismatch at selective interfacial barriers. This design enables optimization of both carrier and phonon at VSe_2_/GeTe interfaces, facilitating carrier‐phonon decoupling and thereby preserving relatively high *µ*_w_ and *S^2^σ*, as shown schematically in Figure 1a. Additionally, the VSe_2_ nanowire precipitates induce intense phonon scattering through phonon frequency mismatch, achieving an ultralow *κ*_l_ (∼0.4 W m^−1^ K^−1^) at 773 K in the (Ge_0.82_Mn_0.04_Bi_0.04_Pb_0.1_Te)_0.99_(VSe_2_)_0.01_. Consequently, the decoupling of carrier and phonon transport by selective interfacial barriers leads to an exceptional ratio of *µ*_w_/*κ*_l_, compared to other typical polycrystalline thermoelectric materials (Figure 1b) [5, 18, 19, 20, 21, 22, 23, 24, 25, 26, 27], culminating in an ultra‐high *ZT* (∼2.7 at 773 K) in the (Ge_0.82_Mn_0.04_Bi_0.04_Pb_0.1_Te)_0.99_(VSe_2_)_0.01_. Moreover, a high *ZT*_ave_ of ∼1.9 (300–773 K) can be obtained in the (Ge_0.82_Mn_0.04_Bi_0.04_Pb_0.1_Te)_0.99_(VSe_2_)_0.01_, surpassing the performance of other typical polycrystalline thermoelectric materials at medium ‐temperature range, such as (Bi, Sb)_2_Te_3_ [18], PbTe [5], PbS [19], PbSe [20], SnSe [23], AgSbTe_2_ [26], SnTe [22], Zintles [24], BiCuSeO [25], Skutterudites [21], and GeTe [27], as shown in Figure 1c.

### 2.2 Semi‐Coherent Interface Characterization

**FIGURE 2:**
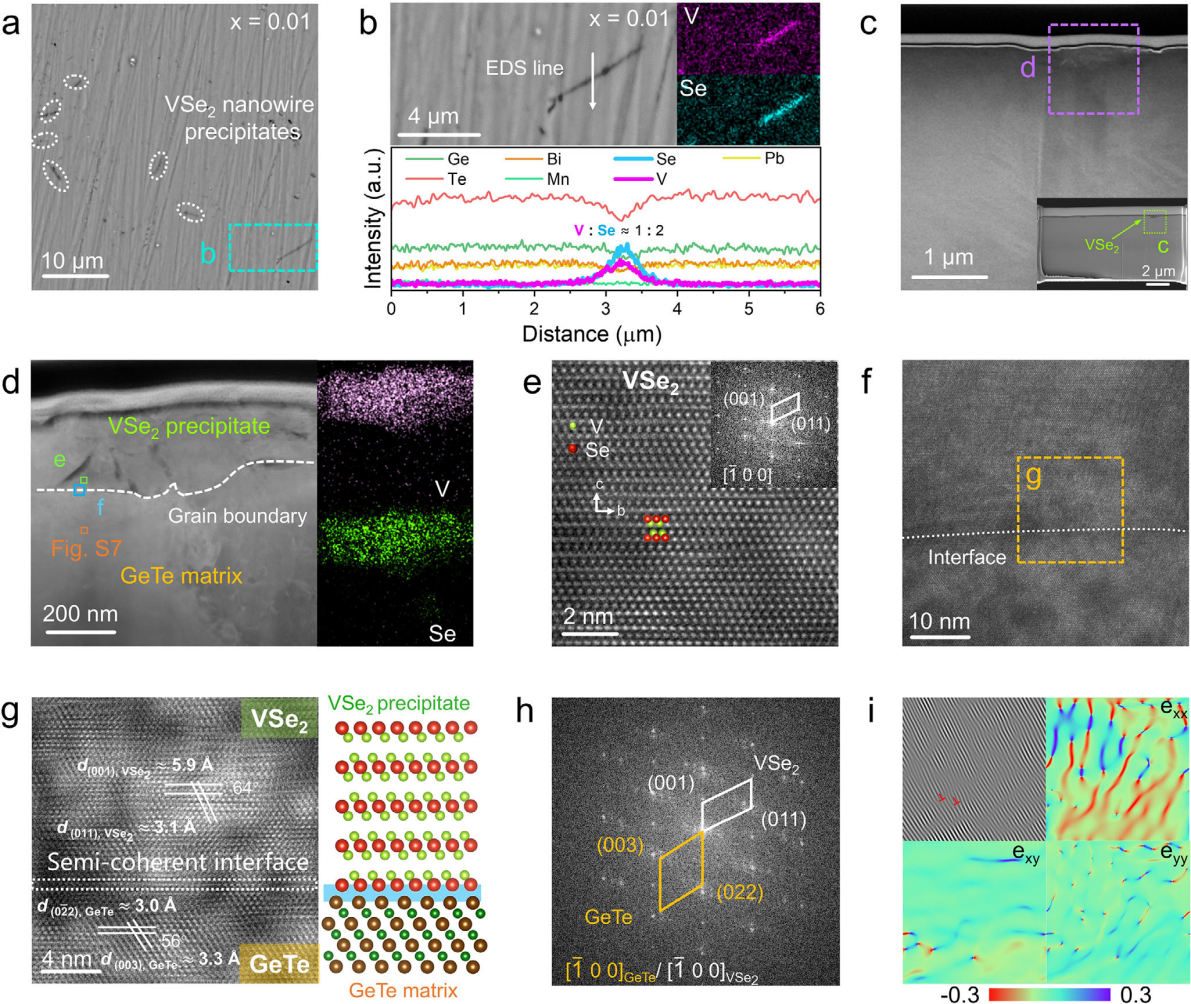
Semi‐coherent interface characterization. (a) Scanning electron microscopy (SEM) image of the (Ge_0.82_Mn_0.04_Bi_0.04_Pb_0.1_Te)_0.99_(VSe_2_)_0.01_ pellet. (b) SEM image (the blue‐squared area of a) with corresponding energy dispersive spectrum (EDS) maps and line. (c) Low‐magnification TEM image of (Ge_0.82_Mn_0.04_Bi_0.04_Pb_0.1_Te)_0.99_(VSe_2_)_0.01_ sample lamella. (d) The enlarged TEM image (the purple area of c) with corresponding EDS. (e) High‐resolution transmission electron microscope (HRTEM) image of the VSe_2_ nanowire precipitate, and the inset is the corresponding Fast Fourier Transform (FFT) pattern. (f) TEM image of the VSe_2_/Ge_0.82_Mn_0.04_Bi_0.04_Pb_0.1_Te interface. (g) HRTEM image of the orange area of (f) and the schematic atomic model of the semi‐coherent interface. (h) Corresponding FFT pattern of the VSe_2_/Ge_0.82_Mn_0.04_Bi_0.04_Pb_0.1_Te interface. (i) IFFT image and corresponding strain mapping of (g).

To understand the structure of the as‐prepared (Ge_0.82_Mn_0.04_Bi_0.04_Pb_0.1_Te)_1‐x_(VSe_2_)_x_ (x = 0‐0.015) samples, the x‐ray diffraction (XRD) was conducted at ambient temperature (Figure S1a). The XRD patterns of all samples can be indexed as R‐GeTe (ICSD‐56042). As the VSe_2_ content increases, the lattice parameter of GeTe remains nearly constant (Figure S1b), indicating the formation of precipitates rather than substitutional or interstitial point defects. No distinct peaks from VSe_2_ are observed, likely due to its low content. Figure 2a exhibits the low‐magnification scanning electron microscopy (SEM) image of the (Ge_0.82_Mn_0.04_Bi_0.04_Pb_0.1_Te)_0.99_(VSe_2_)_0.01_ sample, showing randomly distributed precipitates across the matrix. As the VSe_2_ content increases, the morphology of the precipitate transitions from nanoparticles to nanowires, as illustrated in Figure S2. Figure S3 exhibits a different direction SEM image of (Ge_0.82_Mn_0.04_Bi_0.04_Pb_0.1_Te)_0.99_(VSe_2_)_0.01_ sample. These precipitates possess nanoscale lateral dimensions and a high aspect ratio, and are therefore defined as nanowire precipitates [28]. Meanwhile, the morphology and average width of the VSe_2_ nanowire precipitates in the samples changed significantly with increasing the VSe_2_ content from 0.005 to 0.015, as illustrated in Figure S4. Under a low VSe_2_ content of 0.005, the VSe_2_ precipitates are primarily in the form of nanoparticles, with an average width of approximately 62±20 nm. The extremely low VSe_2_ content provides insufficient driving force for precipitate growth, leading to the formation of small and isolated nanoparticles (Figure S4a,b). When the VSe_2_ content is increased to 0.01, the higher VSe_2_ content enhances the driving force for crystal growth. Meanwhile, adjacent nanoparticles tend to agglomerate to minimize surface energy. Notably, VSe_2_ possesses a layered crystal structure with intrinsic anisotropy, which facilitates the directional agglomeration of nanoparticles. As a result, the morphology evolves into nanowires with an average width of ∼120 ± 30 nm and lengths up to ∼2–4 µm. When the VSe_2_ content is further increased to 0.015, the precipitates continue to grow and coarsen, with widths reaching ∼700 ± 400 nm and lengths up to ∼8 µm, indicating that the VSe_2_ precipitates can no longer maintain the wire morphology. Details of the statistically analysed average width are presented in Figure S4g. The enlarged SEM image with the energy dispersive spectrum (EDS) (Figure 2b; Figure S5) suggests that the precipitates are primarily composed of V and Se with the ratio of ∼1:2, consistent with VSe_2_. A transmission electron microscope (TEM) lamella of the (Ge_0.82_Mn_0.04_Bi_0.04_Pb_0.1_Te)_0.99_(VSe_2_)_0.01_ was prepared using a Focused Ion beam (FIB), showing enriched V and Se areas on the top of the lamella (Figure 2c). TEM‐EDS confirms that the nanowire precipitates are composed solely of V and Se, with no significant inter‐diffusion between the nanowires and matrix (Figure 2d; Figure S6). In addition, it can be observed that the VSe_2_ wire precipitates are assembled from multiple nanoparticles (Figure 2d). The high‐resolution TEM (HRTEM) image of the nanowire precipitate (highlighted by the green square in Figure 2d) is shown in Figure 2e. The lattice structure matches well with the VSe_2_ crystal structure model, indicating that the precipitate is highly crystallized VSe_2_. The inset of Figure 2e shows the Fast Fourier Transform (FFT) pattern, indexed VSe_2_ along the [$\bar{1}$00] zone axis. These findings clearly confirm that the nanowire precipitates are VSe_2_, which aligns with the compositional characterization results.

To better understand interfacial structure features between the VSe_2_ nanowire precipitates and the Ge_0.82_Mn_0.04_Bi_0.04_Pb_0.1_Te matrix, Figure 2f shows an enlarged TEM image of the region outlined in blue in Figure 2d, highlighting the VSe_2_/Ge_0.82_Mn_0.04_Bi_0.04_Pb_0.1_Te interface. Figure 2g shows the HRTEM image of this interface along with the corresponding schematic model. The lattice spacings (*d*) of (011) and (001) plane (i.e., *d*_(011),VSe2,_ and *d*_(001),VSe2_) in VSe_2_ nanowire precipitate are ∼3.1 Å and ∼5.9 Å, respectively. These spacings are integral multiples of *d*_(003), GeTe_ (∼3.4 Å) and *d*_(022), GeTe_ (∼3.0 Å) in the Ge_0.82_Mn_0.04_Bi_0.04_Pb_0.1_Te matrix. The lattice angle between (011) and (001) planes of VSe_2_ nanowire precipitate is 64°, which closely matches the angle between the (003) and ($0\bar{2}2$) planes of the Ge_0.82_Mn_0.04_Bi_0.04_Pb_0.1_Te matrix (56°) (Figure S7). These observations indicate that a clear semi‐coherent interface can be formed between the VSe_2_ nanowire precipitates and the Ge_0.82_Mn_0.04_Bi_0.04_Pb_0.1_Te matrix. Figure 2h shows the FFT pattern of Figure 2e, which further supports the semi‐coherent relationship between the Ge_0.82_Mn_0.04_Bi_0.04_Pb_0.1_Te matrix and the VSe_2_ nanowire precipitates along [$\bar{1}$00] zone axis. Figure 2i shows the IFFT image and corresponding strain mapping of Figure 2g. The semi‐coherent interfaces exhibit a low density of dislocations, which is primarily attributed to the small lattice mismatch (6.25%) between ($0\bar{2}2$) planes of Ge_0.82_Mn_0.04_Bi_0.04_Pb_0.1_Te matrix and (011) planes of VSe_2_ nanowire precipitates. Furthermore, the reduction of defects and dangling bonds near the interface significantly reduces the interfacial strain fields. The successful construction of semi‐coherent interfaces significantly reduced the density of dangling bonds, dislocations, and strain fields at the interface, thus effectively enhancing electron transport.

### 2.3 Optimization of Electron Transport

**FIGURE 3:**
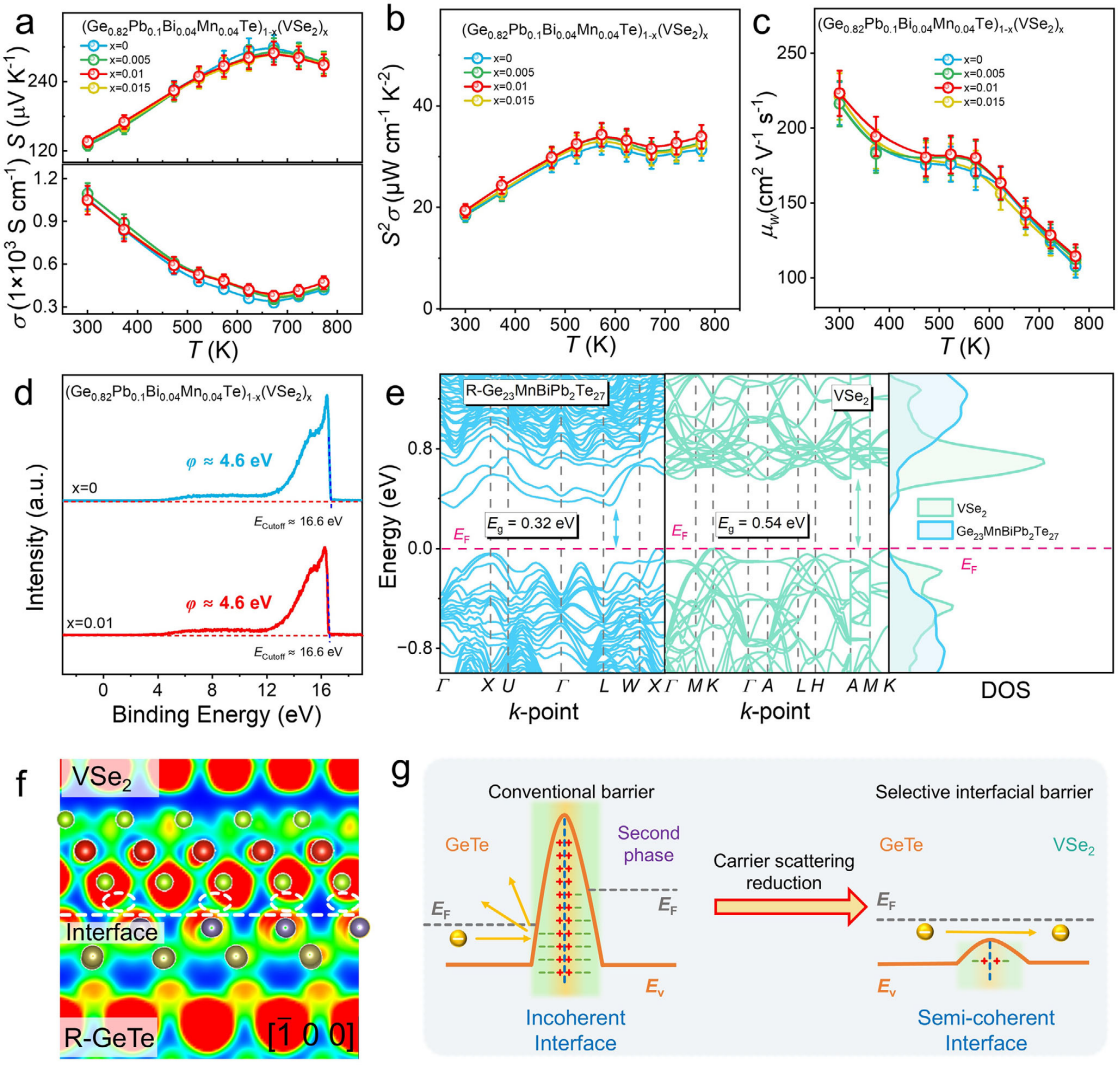
Optimization of electron transport. (a) Temperature‐dependent Seebeck coefficient (*S*) and electrical conductivity (*σ*), (b) power factor (*S^2^σ*), and (c) *µ*_w_ of the (Ge_0.82_Mn_0.04_Bi_0.04_Pb_0.1_Te)_1‐x_(VSe_2_)_x_ (x = 0–0.015) pellets. (d) Ultraviolet photo‐electron spectroscopy plots of the Ge_0.82_Mn_0.04_Bi_0.04_Pb_0.1_Te and (Ge_0.82_Mn_0.04_Bi_0.04_Pb_0.1_Te)_0.99_(VSe_2_)_0.01_ pellets. (e) Band structures calculated for Ge_23_MnBiPb_2_Te_27_ and VSe_2_, with a comparison of the DOS. (f) 2D mappings of ELF for R‐GeTe/VSe_2_ interface. (g) Schematic representation of the comparison between a conventional barrier and a selective interfacial barrier.

Figure 3a presents the temperature‐dependent *σ* and *S* of the as‐prepared (Ge_0.82_Mn_0.04_Bi_0.04_Pb_0.1_Te)_1‐x_(VSe_2_)_x_ (x = 0‐0.015) samples. As shown, the introduction of VSe_2_ nanowire precipitates has only a slight effect on both the *S* and *σ*. Specifically, as the VSe_2_ content increases from 0 to 0.01, *S* shows a slight decrease from 272 to 267 µV K^−1^, while *σ* increases from ∼430 S cm^−1^ to ∼460 cm^−1^ at 773 K. Repeated measurements of the composition‐dependent *n*_h_ for (Ge_0.82_Mn_0.04_Bi_0.04_Pb_0.1_Te)_1‐x_(VSe_2_)_x_ (x = 0–0.015) samples are shown in Figure S8, indicating a slight increase in *n*_h_ with increasing VSe_2_ content. This is attributed to the relatively high intrinsic *n*_h_ of VSe_2_ precipitates dispersed in the matrix [29]. As a result, *S^2^σ* improves from ∼32 to ∼33 µW cm^−1^ K^−2^ slightly after introducing VSe_2_ nanowire precipitates. Figure 3c illustrates the temperature‐dependent *µ*_w_. It can be observed that variations in *µ*_w_ are relatively small and fall within the experimental uncertainty, indicating that the VSe_2_ nanowire precipitates have a minimal impact on carrier scattering. This minimal effect on carrier scattering should be the primary reason for maintaining a high *S^2^σ*.

To better understand the mechanism behind the negligible influence of VSe_2_ nanowire precipitates on carrier scattering, ultraviolet photo‐electron spectroscopy (UPS) was performed to examine the electronic band structure (Figure 3d). The results indicate that the work function (*φ*) of the Ge_0.82_Mn_0.04_Bi_0.04_Pb_0.1_Te and (Ge_0.82_Mn_0.04_Bi_0.04_Pb_0.1_Te)_0.99_(VSe_2_)_0.01_ is approximately ∼4.6 eV, indicating that both Ge_0.82_Mn_0.04_Bi_0.04_Pb_0.1_Te and VSe_2_ have similar *E*_F_. This similarity in *E*_F_ helps reduce carrier scattering at the interface. Furthermore, the band structures of rhombohedral(R)‐Ge_23_MnBiPb_2_Te_27_, cubic(C)‐Ge_23_MnBiPb_2_Te_27,_ and VSe_2_ were calculated using the DFT (Figure 3e; Figure S9). And Figure S10 is the corresponding atomic models of the DFT calculation. The comparison of their electronic band structures and density of states (DOS) suggests that the electronic band structures across the VSe_2_/R‐Ge_23_MnBiPb_2_Te_27_ are well‐aligned. Figure 3f and Figure S11 present 2D mappings of the calculated electron localization function (ELF) for R‐GeTe/VSe_2_ and C‐GeTe/VSe_2_ interfaces. Both VSe_2_/R‐GeTe and VSe_2_/C‐GeTe interface exhibit a highly uniform electron distribution, suggesting that the semi‐coherent interfaces can reduce the dangling bonds and carrier scattering at these interfaces. Figure 3g presents a schematic comparison between a conventional barrier and a selective interfacial barrier. The incoherent interface with the conventional barrier contains a high density of dangling bonds, dislocations, and strain fields. These defects induce high‐density interface states, combined with band mismatch, forming a high interfacial barrier, thus strongly scattering carriers. In contrast, the selective interfacial barriers constructed at the VSe_2_/GeTe interfaces maintain carrier transport, primarily attributed to the significantly reduced interface state density and well‐matched *E*_F_. This synergistic effect effectively suppresses the formation of interfacial barriers, thereby minimizing carrier scattering.

### 2.4 Enhancement of Phonon Scattering and *ZT*

**FIGURE 4:**
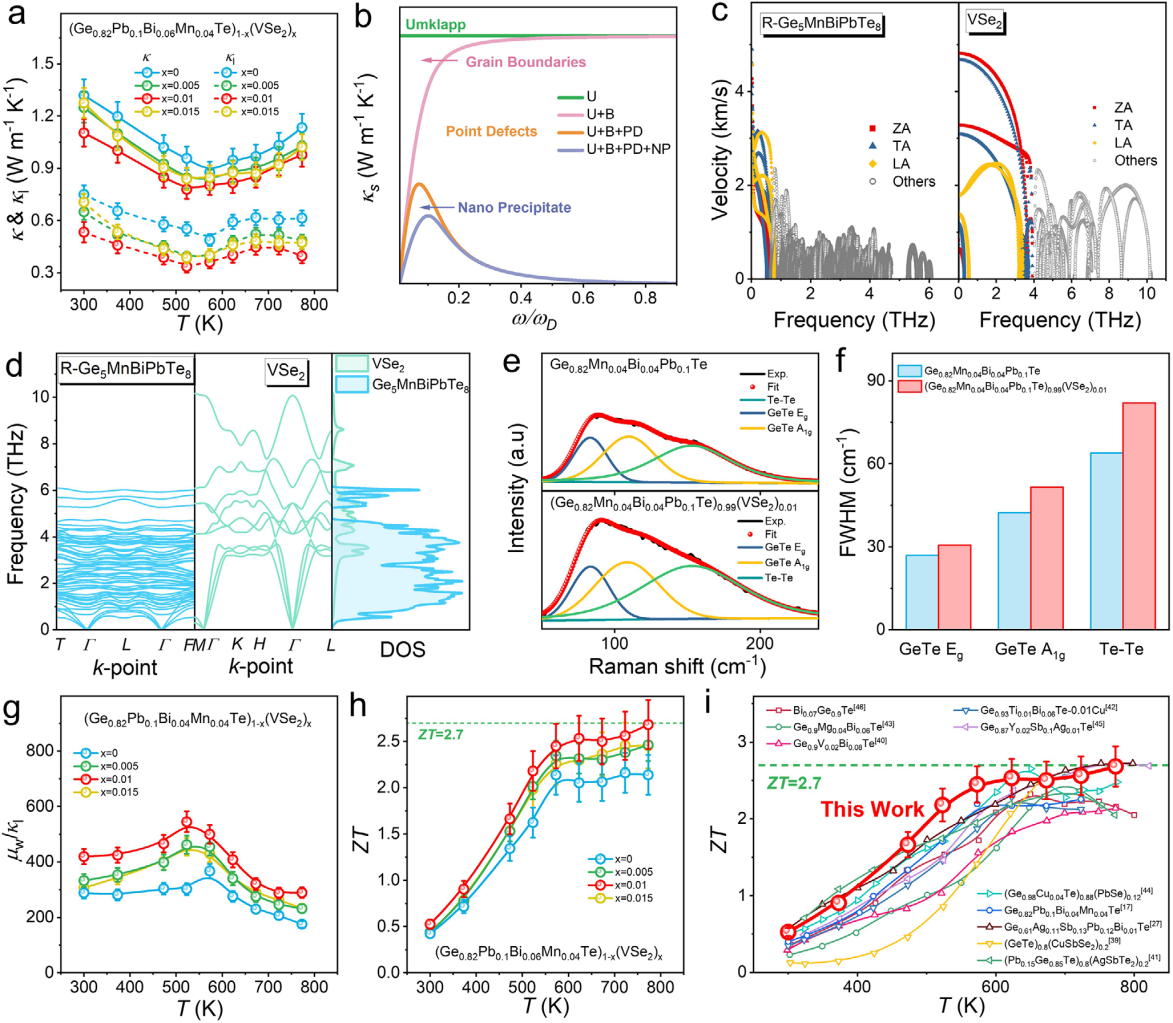
Enhancement of phonon scattering and *ZT*. (a) Temperature‐dependent thermal conductivity (*κ*) and lattice thermal conductivity (*κ*_l_) of the (Ge_0.82_Mn_0.04_Bi_0.04_Pb_0.1_Te)_1‐x_(VSe_2_)_x_ (x = 0–0.015) pellets. (b) Spectral lattice thermal conductivity (*κ*_s_). (c) Phonon group velocity (*v*_g_) of R‐Ge_5_MnBiPbTe_8_ and VSe_2_. (d) Phonon spectra of Ge_5_MnBiPbTe_8_ and VSe_2_, along with a comparison of their phonon DOS. (e) Raman spectra of Ge_0.82_Mn_0.04_Bi_0.04_Pb_0.1_Te and (Ge_0.82_Mn_0.04_Bi_0.04_Pb_0.1_Te)_0.99_(VSe_2_)_0.01_. (f) the Full Width at Half Maximum (FWHM) of the Raman peak. (g) *µ*_w_/*κ*_l_ of the (Ge_0.82_Mn_0.04_Bi_0.04_Pb_0.1_Te)_1‐x_(VSe_2_)_x_ (x = 0–0.015) pellets. (h) Temperature‐dependent *ZT*. (i) High *ZT* achieved for the (Ge_0.82_Mn_0.04_Bi_0.04_Pb_0.1_Te)_0.99_(VSe_2_)_0.01_, compared with other GeTe‐based materials [17, 27, 39, 40, 41, 42, 43, 44, 45, 46].

Figure 4a shows thermal transport properties (*κ* and *κ*_l_) of the material, which experiences a significant reduction after introducing VSe_2_ nanowire precipitates. Specifically, the *κ* reduces from 1.13 W m^−1^ K^−1^ in the Ge_0.82_Mn_0.04_Bi_0.04_Pb_0.1_Te to ∼0.98 W m^−1^ K^−1^ in the (Ge_0.82_Mn_0.04_Bi_0.04_Pb_0.1_Te)_0.99_(VSe_2_)_0.01_ at 773 K. Concurrently, the *κ*_l_ was evaluated by subtracting *κ*_e_ from *κ*, where the 𝜅_e_ can be calculated as 𝜅_e_ = *L𝜎T* with *L* as the Lorenz number evaluated by *L* = 1.5+exp [$-\frac{\left|S\right|}{116}$] [30]. And the temperature‐dependent 𝜅_e_ and *L* are shown in Figure S12a,b. Following the introduction of VSe_2_ nanowire precipitates, the *κ*_l_ drops to an ultralow value of ∼0.4 W m^−1^ K^−1^ in the (Ge_0.82_Mn_0.04_Bi_0.04_Pb_0.1_Te)_0.99_(VSe_2_)_0.01_ at 773 K. However, it can be observed that the *κ*_l_ gradually improves from 0.4 to 0.47 W m^−1^ K^−1^ as the VSe_2_ content increases to 0.015. This phenomenon is attributed to the growth of VSe_2_ nanowire precipitates from nanowires with an average width of ∼120±30 nm into micrometer precipitates with the size of ∼700±400 nm, leading to a reduction in scattering centers for mid‐frequency phonons. Besides the size effect of the VSe_2_ precipitates, the increase in overall *κ*_l_ can be partially attributed to the growing contribution of high *κ*_l_ VSe_2_ precipitate [29].

To better understand the ultralow *κ*_l_ of the (Ge_0.82_Mn_0.04_Bi_0.04_Pb_0.1_Te)_0.99_(VSe_2_)_0.01_, the spectral lattice thermal conductivity (*κ*_s_) was calculated as shown in Figure 4b. As can be seen, low‐ and high‐frequency phonons are predominantly scattered by grain boundaries (B) and point defects (PD), respectively. The introduction of nanoprecipitates results in more effective scattering of mid‐frequency phonons. The combination of various defects leads to a hierarchical phonon scattering effect. To further investigate the mechanism behind the ultralow *κ*_l_, we calculated the phonon group velocity (*v*_g_) of VSe_2_ and Ge_5_MnBiPbTe_8_ using the DFT calculation (Figure 4c). The results reveal that the *v*_g_ of VSe_2_ is significantly higher than that of Ge_5_MnBiPbTe_8_, indicating that the observed ultralow *κ*_l_ in (Ge_0.82_Mn_0.04_Bi_0.04_Pb_0.1_Te)_0.99_(VSe_2_)_0.01_ is not a result of introducing nanoprecipitates with inherently low *v*_g_. To gain further insights, we systematically analysed the phonon dispersion curves and DOS of VSe_2_ and Ge_5_MnBiPbTe_8_ to understand the phonon transport modes across their interface (Figure 4d). The phonon spectrum of Ge_5_MnBiPbTe_8_ shows a continuous dispersion in the low‐frequency region, with a pronounced DOS peak, indicating that phonon transport in this material predominantly occurs through low‐frequency channels. In contrast, VSe_2_ displays a much weaker DOS peak in the same region. Additionally, the phonon frequency distribution of VSe_2_ partially complements the phonon gap in Ge_5_MnBiPbTe_8_, its high‐frequency modes also extend far beyond the phonon spectrum of Ge_5_MnBiPbTe_8_. This results in a significantly reduced overlap of their phonon DOS (*S*_DOS_). The *S*_DOS_ can indicate the heat transfer of the interface of the two materials [31], which can be expressed as: $S_{DOS}=\frac{\int_{0}^{\infty }F_{1}\left(w\right)F_{2}\left(w\right)dw}{\int_{0}^{\infty }F_{1}\left(w\right)dw*\int_{0}^{\infty }F_{2}\left(w\right)dw}$. It can be noteworthy that the *S*_DOS_ dramatically reduces to 0.012 (VSe_2_/Ge_5_MnBiPbTe_8_ interface), as shown in Figure 1a. Therefore, the low *S*_DOS_ of VSe_2_/Ge_5_MnBiPbTe_8_ interfaces and the high difference of *v*_g_ between Ge_5_MnBiPbTe_8_ and VSe_2_ indicate the formation of an effective phonon scattering at the interface. These selective interfacial barriers allow only a minimal number of phonons to traverse the interface while causing the majority of phonons to be scattered. In addition, we further calculated the phonon spectra of C‐Ge_5_MnBiPbTe_8_ (Figure S14) and found no significant difference between the phonon spectra of C‐Ge_5_MnBiPbTe_8_ and R‐Ge_5_MnBiPbTe_8_. This result indicates that the interface scattering of phonons caused by phonon frequency mismatch can be effectively maintained after the phase transition.

To clarify the phonon scattering at Ge_5_MnBiPbTe_8_/VSe_2_ interface, Figure 4e evaluates the thermal transport by the lifetime of Raman optical phonon *τ*, *τ* ∝1/*Γ* (*Γ* is the Full Width at Half Maximum, FWHM) [32], which is widely used to reflect the *κ*_l_ [33, 34, 35]. As can be seen, three peaks can be observed at 83, 109, and 152 cm^−1^ for Ge_0.82_Mn_0.04_Bi_0.04_Pb_0.1_Te and (Ge_0.82_Mn_0.04_Bi_0.04_Pb_0.1_Te)_0.99_(VSe_2_)_0.01_. The peaks at 83 cm^−1^ (doubly degenerate *E_g_* symmetry optical/longitudinal optical mode) and 109 cm^−1^ (A_1_g symmetry optical mode) originate from Ge─Te bond vibrations [33], while the 152 cm^−1^ peak arises from long‐range Te–Te interactions. Figure 4f shows the FWHM of Ge_0.82_Mn_0.04_Bi_0.04_Pb_0.1_Te and (Ge_0.82_Mn_0.04_Bi_0.04_Pb_0.1_Te)_0.99_(VSe_2_)_0.01_. As can be seen, the FWHM of (Ge_0.82_Mn_0.04_Bi_0.04_Pb_0.1_Te)_0.99_(VSe_2_)_0.01_ increases significantly with the introduction of the nanowire VSe_2_ precipitates, confirming that Ge_0.82_Mn_0.04_Bi_0.04_Pb_0.1_Te /VSe_2_ interface can effectively reduce the phonon lifetime. Therefore, the phonon scattering caused by phonon frequency mismatch significantly suppresses the efficiency of cross‐interface phonon transport, resulting in a notable enhance in Kapitza resistance at the interface and reducing the phonon lifetime, ultimately resulting in a significant reduction in *κ*_l_ [36, 37, 38].

The selective interfacial barriers enable effective decoupling of carrier and phonon scattering, leading to an obviously increase in *µ*_w_/*κ*_l_ (Figure 4g). This process results in an enhanced *ZT*, with a peak value of ∼2.7 at 773 K for the (Ge_0.82_Mn_0.04_Bi_0.04_Pb_0.1_Te)_0.99_(VSe_2_)_0.01_, up from 2.2 in the pure Ge_0.82_Mn_0.04_Bi_0.04_Pb_0.1_Te (Figure 4h). It can be noted that as the VSe_2_ content increases to 0.015, the *ZT* value decreases from ∼2.7 to ∼2.5 due to the enhancement of *κ*_l_. Therefore, a high plateaued *ZT* greater than 2.3 was observed between 573 and 773 K (Figure 4i), leading to a record‐high *ZT*_ave_ of 1.9 across 300–773 K (Figure 1d), compared to the other state‐of‐the‐art GeTe‐based materials [17, 27, 39, 40, 41, 42, 43, 44, 45, 46].

In addition, high repeatability and reproducibility of this performance are documented in Figures S15 and S16. Figure S17 presents the third‐party measurements of electrical and thermal properties, and Figure S18 provides a comparison with our results, supporting the reliability of our reported high *ZT* values. Figure S19 presents the thermoelectric properties of (Ge_0.82_Mn_0.04_Bi_0.04_Pb_0.1_Te)_0.99_(VSe_2_)_0.01_ along different directions, suggesting that the polycrystalline GeTe exhibits weak anisotropy, which further supports the reliability of the high *ZT* value. The thermogravimetric analysis (TGA) and Vickers hardness test results (Figure S20) demonstrate excellent thermal stability and mechanical performance. In addition, XRD and SEM analyses of the (Ge_0.82_Mn_0.04_Bi_0.04_Pb_0.1_Te)_0.99_(VSe_2_)_0.01_ sample after thermal cycling tests (Figures S21 and S22) exhibit no shift in diffraction peaks, no emergence of new peaks, and no change in nanowire diameter. These provide direct evidence that no phase decomposition or microstructural coarsening occurred at the operating temperature, further confirming the reproducibility and reliability of the high *ZT* we reported.

### 2.5 Power Generation

**FIGURE 5:**
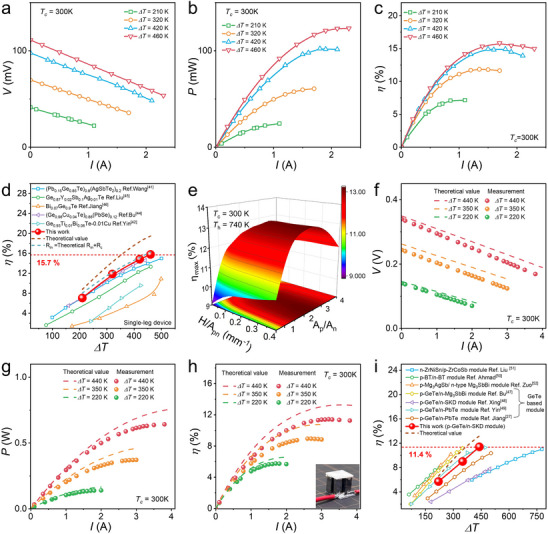
Performance of the GeTe‐based device. The (a) voltage (*V*), (b) output power (*P*), and (c) conversion efficiency (*η*) of the single‐leg device as a function of current (*I*). (d) Maximum *η* of the single‐leg device at various temperature differences (∆*T*), compared to the previously reported results for other GeTe‐based single‐leg devices [41, 42, 44, 45, 46]. (e) Optimization of the dimensional structure of the GeTe‐based module by simulation. (f) *I*‐dependent *V* and (g) *P* for the GeTe‐based module, compared with theoretical predictions to validate performance. (h) *I*‐dependent *η* of the module, evaluated against theoretical expectations to assess model accuracy and areas for optimization. (i) Maximum *η* for the GeTe‐based module under ∆*T*, benchmarked against other GeTe‐based modules reported in the literature [27, 47, 48, 49, 50, 51, 52].

To validate the ultra‐high thermoelectric performance of the (Ge_0.82_Mn_0.04_Bi_0.04_Pb_0.1_Te)_0.99_(VSe_2_)_0.01_ sample, a single‐leg device was fabricated. The voltage (*V*) and output power (*P*) under varying temperature difference (∆*T*) were measured as a function of current (*I*) (Figure 5a,b). It was observed that the *V* increased from 41.4 to 111.1 mV as the ∆*T* increased from 210 to 460 K, which closely matched the simulated results based on the material performance (Figure S23a). As can be seen, the maximum *P* increased from 24 mW at a *∆T* of 210 K to 123 mW at 460 K. However, this value was lower than the theoretical maximum due to the presence of additional contact resistance (Figure S23b). Despite this, the device exhibited a maximum *η* of ∼15.7% at a *∆T* of 460 K (Figure 5c), which is among the highest efficiencies recorded for GeTe‐based single‐leg devices (Figure 5d) [41, 42, 44, 45, 46]. The difference between the experimental and theoretical maximum efficiency is attributed to the additional contact resistance (*R*_c_), which results in a lower output power than the theoretical value.

To clarify the *R*_c_ of the as‐prepared single‐leg device, Figure S24 plots resistance line scanning across the interfaces of the (Ge_0.82_Mn_0.04_Bi_0.04_Pb_0.1_Te)_0.99_(VSe_2_)_0.01_/Ni/Ag leg. As can be seen, the *R*_c_ between the Ni barrier layer and (Ge_0.82_Mn_0.04_Bi_0.04_Pb_0.1_Te)_0.99_(VSe_2_)_0.01_ reaches approximately ∼0.7 mΩ. This relatively high *R*_c_ is attributed to the insufficient interfacial bonding strength between the Ni and the (Ge_0.82_Mn_0.04_Bi_0.04_Pb_0.1_Te)_0.99_(VSe_2_)_0.01_. In addition, we included the measured *R*_c_ into Finite Element Analysis (FEA) for computational simulation (defined as *R*_in_ = theoretical *R*_in_+ *R*_c_) and systematically compared the results with both theoretical predictions and experimental measurements. The results show that theoretical values exhibit significantly better agreement with the experimental data when *R*_c_ is taken into account, highlighting the importance of including *R*_c_ in accurately modeling device performance.

To demonstrate the thermoelectric performance, a *π*‐type module was assembled using two pairs of p‐n junctions with *p*‐type (Ge_0.82_Mn_0.04_Bi_0.04_Pb_0.1_Te)_0.99_(VSe_2_)_0.01_ and n‐type S_0.26_Co_4_Sb_11.11_Te_0.73_. Figure S25 plots the corresponding performance of the n‐type materials. To optimize the performance of the module, an FEA was conducted to determine the optimal geometric configuration (Figure 5e), suggesting that the optimal area ratio of p‐type to n‐type legs (*A*_p_/*A*_n_) is ∼1.9 and the ratio of the height and cross‐sectional area (*H*/*A*_pn_) is 0.4. Considering fabrication constraints, the device was constructed with *A*_p_/*A*_n_ ≈ 1.78 and *H/A*_pn_ ≈ 0.32. Specifically, the p‐ and n‐type legs were cut to dimensions of 4 × 4 × 8 mm^3^ and 3 × 3 × 8 mm^3^, respectively. The calculated *η* at the actual geometric configuration (∼13.1%) is very close to the theoretical maximum (∼13.4%), indicating that such deviations have a negligible impact on device performance. As shown in Figure 5f, the measured *V* of the π‐type thermoelectric module closely matched the theoretical *V* across *ΔT* ranging from 220 to 440 K. However, the additional resistance within the system resulted in the *P* and *η* lower than theoretical predictions. It should be noted that all simulations were performed based on the actual geometric dimensions and measured transport parameters of the fabricated device. Therefore, we included the measured *R*_c_ into FEA for computational simulation. It can be seen that the results show excellent agreement between the simulated and experimentally measured values, as shown in Figure S26. Despite these losses, the *π*‐type thermoelectric module was able to achieve an exceptionally high *P* (0.64 W) and *η* (11.4%) at a *ΔT* of 440 K, respectively (Figure 5g,h). The inset of Figure 5h is an optical image of the *π*‐type thermoelectric module. The *η* achieved by the π‐type thermoelectric module is relatively high among GeTe‐based [27, 47, 48, 49] and other material systems [50, 51, 52] thermoelectric modules without segmentation, as shown in Figure 5i. This outstanding performance is primarily attributed to the high efficiency of the p‐type (Ge_0.82_Mn_0.04_Bi_0.04_Pb_0.1_Te)_0.99_ (VSe_2_)_0.01_.

## 3 Conclusion

Our study reveals that selective interfacial barriers caused by introducing VSe_2_ nanowire precipitates play a pivotal role in enhancing *µ*_w_/*κ*_l_ and thermoelectric performance by effectively carrier‐phonon decoupling. Specifically, this design effectively minimizes *E*_F_ mismatch by the similar *E*_F_ of VSe_2_ and matrix, while significantly decreasing interface state density induced by dangling bonds, dislocations, and strain fields via semi‐coherent interface. These combined effects significantly suppress interface potential electron fluctuations and barriers, thereby enabling high *µ*_w_. Concurrently, phonon frequency mismatch establishes an effective phonon scattering that strongly inhibits phonon transport, resulting in ultralow *κ*_l_ of ∼0.4 W m^−1^ K^−1^. The selective interfacial barriers yield an ultra‐high peak *ZT* (∼2.7) and *ZT*_ave_ (∼1.9). As validation, a π‐type thermoelectric module was constructed, which achieved a high *η* of ∼11.4% at a *ΔT* of 440 K. This work underscores the potential of selective interfacial barriers for enhancing thermoelectric performance and advancing waste heat recovery technologies.

## Funding

This work was supported by the National Natural Science Foundation of China (No. 52272040); State Key Laboratory of Materials‐Oriented Chemical Engineering (SKL‐MCE‐23A04, SKL‐MCE‐25A02); Priority Academic Program Development of Jiangsu Higher Education Institutions (PAPD); Jiangsu Specially‐Appointed Professor Program Postgraduate Research & Practice Innovation Program of Jiangsu Province. ZGC thanks the finacial support from the Australian Research Council, and the QUT Capacity Building Professor Program; National Computational Infrastructure supported by the Australian government (Project wk98).

## Conflicts of Interest

The authors declare no conflicts of interest.

## Supporting information




**Supporting File**: advs75403‐sup‐0001‐SuppMat.docx.

## Data Availability

The data that support the findings of this study are available from the corresponding author upon reasonable request.
