# Supplementary material for: Selective Interfacial Barriers Drive High‐Performance GeTe Thermoelectrics
Source: Adv Sci (Weinh). 2026 Apr 20;13(40):e75403. doi: 10.1002/advs.75403 (PMC13335450; doi:10.1002/advs.75403)
Supplement: Supplementary file 1 — Supporting File: advs75403‐sup‐0001‐SuppMat.docx. [file ADVS-13-e75403-s001.docx]

Supporting Information

Selective Interfacial Barriers Drive High-Performance GeTe Thermoelectrics

*Liang-Cao Yin,* *Xinhua Lu, Wei-Di Liu*, Meng Li, Siqi Liu, De-Zhuang Wang, Hao Wu, Yuan-Meng Liu, Xiao-Lei Shi, Yifeng Wang, Lixiong Zhang, Qingfeng Liu*, and Zhi-Gang Chen**

Prof. Q. F. Liu, Prof. L. X. Zhang, Mr. L.-C. Yin, Mr. X. Lu, Mr. D.-Z. Wang, Mr. H. Wu, Miss. Y.-M. Liu

State Key Laboratory of Materials-Oriented Chemical Engineering, College of Chemical Engineering, Nanjing Tech University, Nanjing 211816, China.

Email: [qfliu@njtech.edu.cn](mailto:qfliu@njtech.edu.cn)

Prof. Z.-G. Chen, Dr. W.-D. Liu, Dr. M. Li, Mr. S.-Q. Liu, Dr. X.-L. Shi

School of Chemistry and Physics, ARC Research Hub in Zero-emission Power Generation for Carbon Neutrality, and Center for Materials Science, Queensland University of Technology, Brisbane, QLD 4000, Australia.

E-mail: [weidi.liu@qut.edu.au](mailto:weidi.liu@qut.edu.au)

E-mail: [zhigang.chen@qut.edu.au](mailto:zhigang.chen@qut.edu.au)

Prof. Y. F. Wang

College of Materials Science and Engineering, Nanjing Tech University, Nanjing 211816, China.

Keywords: GeTe, thermoelectric, figure-of-merit, carrier-phonon decoupling

**Methods**

**Rare materials** Ge (pieces, 99.999 %, Chengdu optoelectronic materials Co., Ltd.), Te (pieces, 99.999 %, Chengdu optoelectronic materials Co., Ltd.), Se (pieces, 99.99 %, Chengdu optoelectronic materials Co., Ltd.), Pb (pieces, 99.99 %, Aladdin), Mn (powder, 99.99 %, Aladdin), Bi (pieces, 99.99 %, Aladdin), V (powder, 99.99 %, Aladdin).

**Synthesis** (Ge_0.82_Mn_0.04_Bi_0.04_Pb_0.1_Te)_1-x_(VSe_2_)_x_ (x=0-0.015) samples were synthesized by melting in evacuated and carbon-coated silica tubes at nominal stoichiometry. For example, in the 3-g sample, the relative weighing errors for elements with smaller amounts, such as V, Se, and Mn, are controlled within 1%, while for elements with larger quantities, including Ge, Te, Bi, and Pb, the relative errors are strictly maintained within 0.2%. The silica tubes were heated up to 1223 K for 12h and held at this temperature for 20 h before being quenched in ice water. The cooled silica tubes were annealed at 923 K for 3 days. The acquired ingots of (Ge_0.82_Mn_0.04_Bi_0.04_Pb_0.1_Te)_1-x_(VSe_2_)_x_ were pulverized into powder by an agate mortar for 20 min, and then consolidated by spark plasma sintering (SPS, LABOX-110H Sinter Land). In the sintering process, the powder was loaded into a graphite mold, placed into the furnace chamber, and evacuated. The pressure was initially set to 16.5 MPa, and the heating rate was set to 60 K/min. When the temperature reached 803 K, the pressure increased to 55 MPa. Upon reaching 823 K, the temperature was maintained for 5 minutes (with temperature fluctuations controlled within ±1 K). Then, the sample was allowed to cool naturally.

**Characterization** The phase structure of (Ge_0.82_Mn_0.04_Bi_0.04_Pb_0.1_Te)_1-x_(VSe_2_)_x_ (x=0-0.015) was analyzed by XRD (Smartlab) with Cu K_α_ radiation at the room temperature. The work function of materials was confirmed by UPS (Thermo ESCALAB 250, Japan) with a He I*α* radiation source. The work function (*φ*) can be determined from the equation *φ*= *hν* - *E*_cutoff_. The microstructure and composition of fabricated pellets were revealed by SEM (Hitachi TM3000, Japan) equipped with EDS and TEM (FEI Talos F200S). The (Ge_0.82_Mn_0.04_Bi_0.04_Pb_0.1_Te)_0.99_(VSe_2_)_0.01_ lamella for TEM characterization was cut by Focused Ion beam. Thermogravimetric analysis (TGA, STA449F3, NETZSCH) of Ge_0.82_Mn_0.04_Bi_0.04_Pb_0.1_Te and (Ge_0.82_Mn_0.04_Bi_0.04_Pb_0.1_Te)_0.99_(VSe_2_)_0.01_ samples were performed from 323 to 773 K with a heating rate of 10 K min^−1^. The Vickers hardness was measured three times for each sample by using a Vickers hardness test system (Wilson Tukon1102, America). Raman spectroscopy (Labram HR800, Horibra) was conducted with a 514 nm laser source in the range from 50 to 300 cm^-1^ at room temperature.

**Thermoelectric Performance Measurement** The temperature-dependent *σ* and *S* from 300 to 773 K were measured simultaneously under Argon atmosphere by a commercial four-probe measuring system (SBA 458, NETZSCH, Germany and ZEM-3, Advance Riko, Japan). Based on *κ* was calculated by *κ* = *D* × *C*_p_ × *ρ*, where thermal diffusivity (*D*) was measured based on a laser flash method (LFA 457 and LFA 467, NETZSCH, Germany), heat capacity (*C*_p_) values were calculated via the Dulong-Petit’s law. The experimental *C*_p_ of (Ge_0.82_Mn_0.04_Bi_0.04_Pb_0.1_Te)_0.99_(VSe_2_)_0.01_ sample was measured using a differential scanning calorimetry thermal analyzer (DSC, DSC2500, TA). The measured value is in close agreement with the theoretical prediction (Figure S27). The density (*ρ*) was determined by the Archimedes method, and the *ρ* is as shown in Table S1.

**Error Bar Determination** Herein the error bars (Table S2) were labelled in each temperature dependent thermoelectric parameter plots to describe the uncertainties of measurement. The uncertainties of directly measured values were determined based on the deviation among several measurements. Eventually, the uncertainties of indirectly measured values could be calculated:

$$\varepsilon\left( s \right)=\sum_{i=1}^{n} \left( s_{i}-\bar{s} \right)/n\bar{s}$$

$$\varepsilon\left( \sigma\right)=\sum_{i=1}^{n} \left( \sigma_{i}-\bar{\sigma} \right)/n\bar{\sigma}$$

$$\varepsilon\left( \kappa\right)=\sum_{i=1}^{n} \left( \kappa_{i}-\bar{\kappa} \right)/n\bar{\kappa}$$

$$2\varepsilon\left( PF \right)=\surd\bar{4\left[ 2\varepsilon\left( s \right) \right]^{2}+\left[ 2\varepsilon\left( \sigma\right) \right]^{2}}$$

$$2\varepsilon\left( ZT \right)=\surd\bar{\left[ 2\varepsilon\left( PF \right) \right]^{2}+\left[ 2\varepsilon\left( \kappa\right) \right]^{2}}$$

**Hall measurement** The *n*_h_ of (Ge_0.82_Mn_0.04_Bi_0.04_Pb_0.1_Te)_1-x_(VSe_2_)_x_ (x=0-0.015) were estimated by *n*_h_=1/eR_H_, where *R*_H_ is the Hall coefficient. And the *R*_H_ values at room temperature were measured by the Van der Pauw method (CH-70, CH-magnetoelectricity Technology Co., Ltd., China) under a magnetic field up to 500 mT.

**Density Functional Theory Calculations** DFT calculations were performed using the all electron projected augmented wave (PAW) method, as implemented in the Vienna Ab initio Simulation Package (VASP). The generalized gradient approximation (GGA) with the fully relativistic Perdew-Burke-Ernzerhof (PBE) functional was employed to treat the exchange correlations.^[1]^ The valence wave functions were expanded in a plan-wave basis with a cut-off energy of 450 eV. All atoms were allowed to relax in their geometric optimizations until the Hellmann–Feynman force is less than 1×10^–3^ eV·Å^–1^. The convergence criterion and the Monkhorst-Pack **k**-mesh adopted for ionic relaxation are 1×10^–7^ eV per electron and 0.03 per Å^3^, respectively. A denser 0.02 Å^–3^ Monkhorst-Pack **k**-mesh was adopted for calculating DOS, and a line-mode **k**-path based on Brillouin path features indicated by the AFLOW framework was adopted for calculating band structures^[2]^. To precisely predict bandgap, the Hubbard U model was considered, with the on-site coulombic (U) and the exchange (J) terms combined in a single effective U parameter of 3.25 eV for V_3d orbitals. Spin-orbital coupling (SOC) was considered because Te is a heavy element. The phononic band structures were calculated along the same **k**-path using Phonopy software package.

**Module Fabrication and Measurements of Thermoelectric Efficiency** (Ge_0.82_Mn_0.04_Bi_0.04_Pb_0.1_Te)_0.99_(VSe_2_)_0.01_ and S_0.26_Co_4_Sb_11.11_Te_0.73_ were used as p-type and n-type legs to assemble a module, respectively. The Ni and Ag was used as a diffusion barrier and contact layer and consolidated by electrochemical plating technology, respectively. On this basis, the p- and n-type legs were designed with dimensions of 4 × 4 × 8 mm^3^ and 3 × 3 × 8 mm^3^, respectively. The p- and n-type legs were cut by wire cutting system. The legs were then connected with the silver-coated aluminium nitride by soldering directly. The hot and cold side of module was connected by sintered silver paste (sintering point of 250 ℃ and melting point of 900 ℃, XY-ASP-N250, Guangzhou Xianyi Electronic Technology Co., LTD). The single-leg device with typical dimensions of 3× 3 × 9 mm^3^ was cut by wire cutting system. And the welding method of the single-leg device was the same as the module. Power generating performance of the single-leg device and module were measured by a home-made system (and the details are shown in Figure S28), where the cold-side temperature is limited to 300 K by cycling water. The *R*_c_ is measured by fixing the device on a four-probe test bench (CM-4, Shenzhen Cindbest Technology CO..LTD), changing the probe positions sequentially, and measuring the corresponding resistance values by the source Meter (Keithley2450).

**Finite Element Analysis** The geometric configuration optimization of the (Ge_0.82_Mn_0.04_Bi_0.04_Pb_0.1_Te)_0.99_(VSe_2_)_0.01_/ S_0.26_Co_4_Sb_11.11_Te_0.73_ thermoelectric module was performed to improve the thermoelectric conversion efficiency. Based on the thermoelectric performance of materials (including the *σ*, the *S* and the total thermal conductivity *κ*), we built a thermoelectric model of a π-type thermoelectric module with 2-pair p-n junctions. We first changed the *A*_p_/*A*_n_, and then adjusted the *H* of module. The result of simulation as shown in Figure 5e. And the model of module performance simulation was shown in Figure S29.


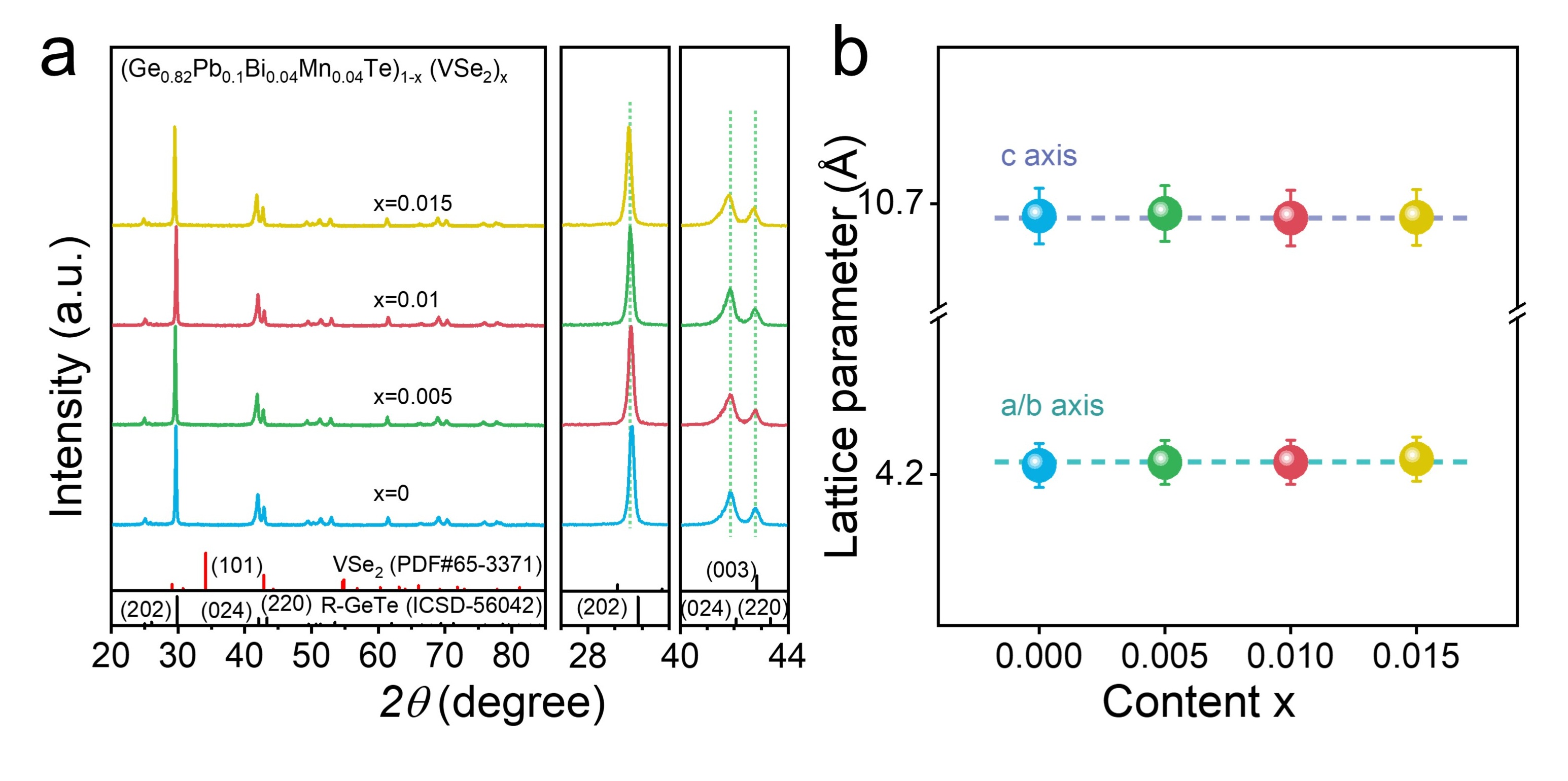


Figure S1. Phase structure characterization of (Ge_0.82_Mn_0.04_Bi_0.04_Pb_0.1_Te)_1-x_(VSe_2_)_x_ (x=0-0.015) samples. (a) X-ray diffraction (XRD) patterns of the as-sintered (Ge_0.82_Mn_0.04_Bi_0.04_Pb_0.1_Te)_1-x_(VSe_2_)_x_ (x=0-0.015) samples. (b) Experimental lattice parameter of (Ge_0.82_Mn_0.04_Bi_0.04_Pb_0.1_Te)_1-x_(VSe_2_)_x_ (x=0-0.015) samples.


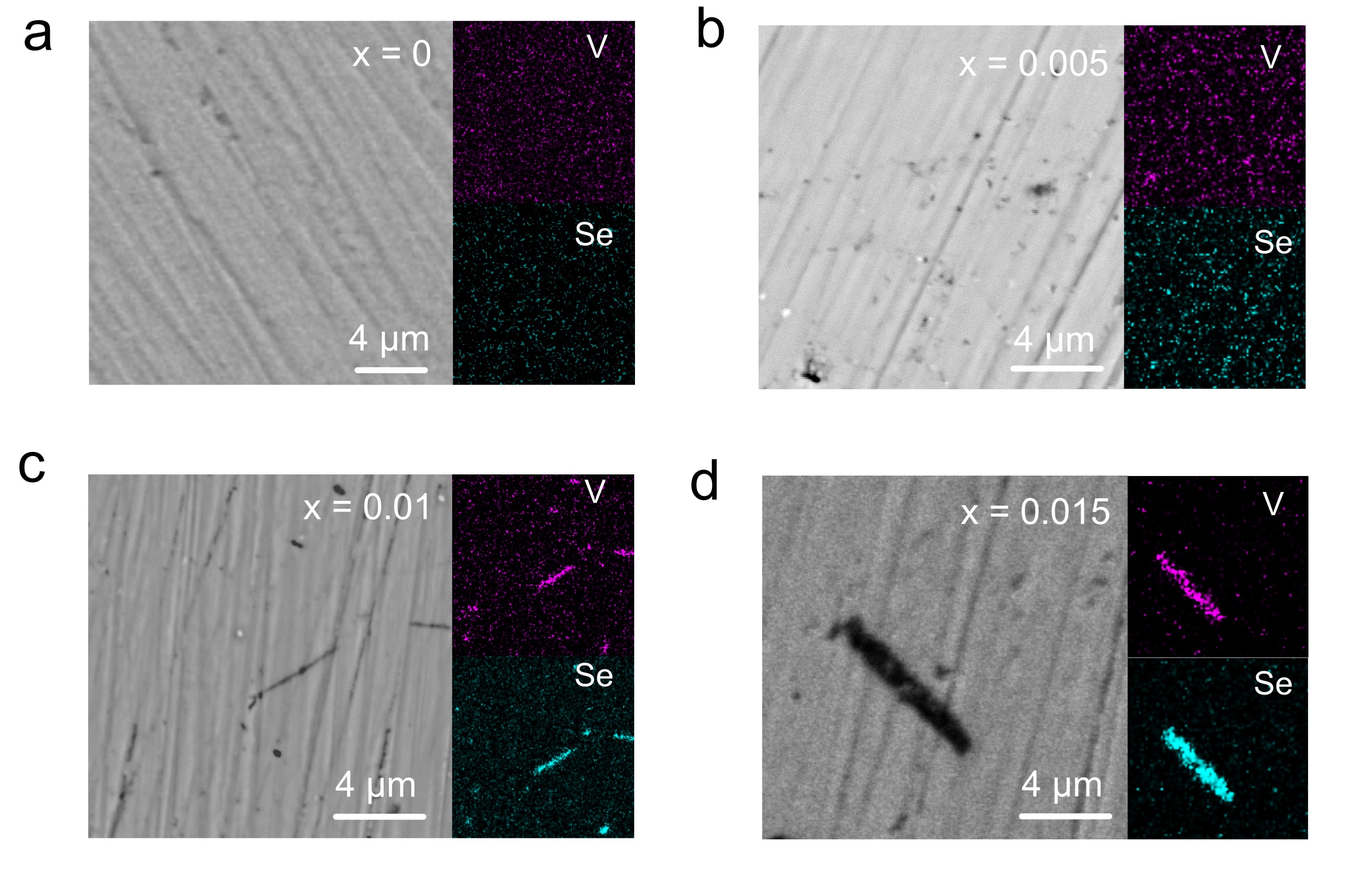


Figure S2. Morphology evolution of VSe_2_ nanoprecipitates. Scaning electron microscopy (SEM) images with the corresponding energy dispersive spectrum (EDS) of (a) Ge_0.82_Mn_0.04_Bi_0.04_Pb_0.1_Te, (b) (Ge_0.82_Mn_0.04_Bi_0.04_Pb_0.1_Te)_0.995_(VSe_2_)_0.005_, (c) (Ge_0.82_Mn_0.04_Bi_0.04_Pb_0.1_Te)_0.99_(VSe_2_)_0.01_, (d) (Ge_0.82_Mn_0.04_Bi_0.04_Pb_0.1_Te)_0.985_(VSe_2_)_0.015_.


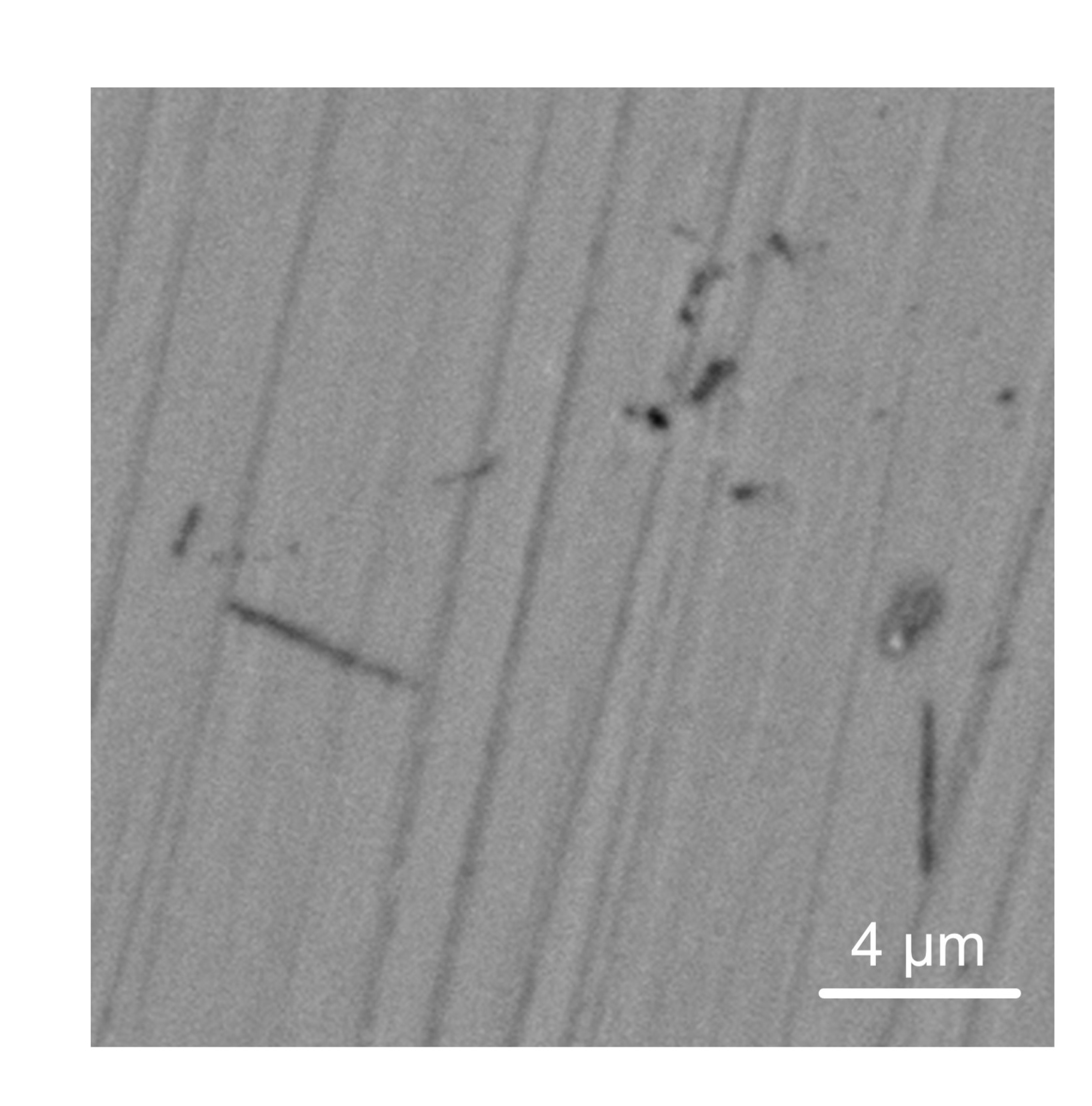


Figure S3. The out of plane direction SEM image of (Ge_0.82_Mn_0.04_Bi_0.04_Pb_0.1_Te)_0.99_(VSe_2_)_0.01_ sample.


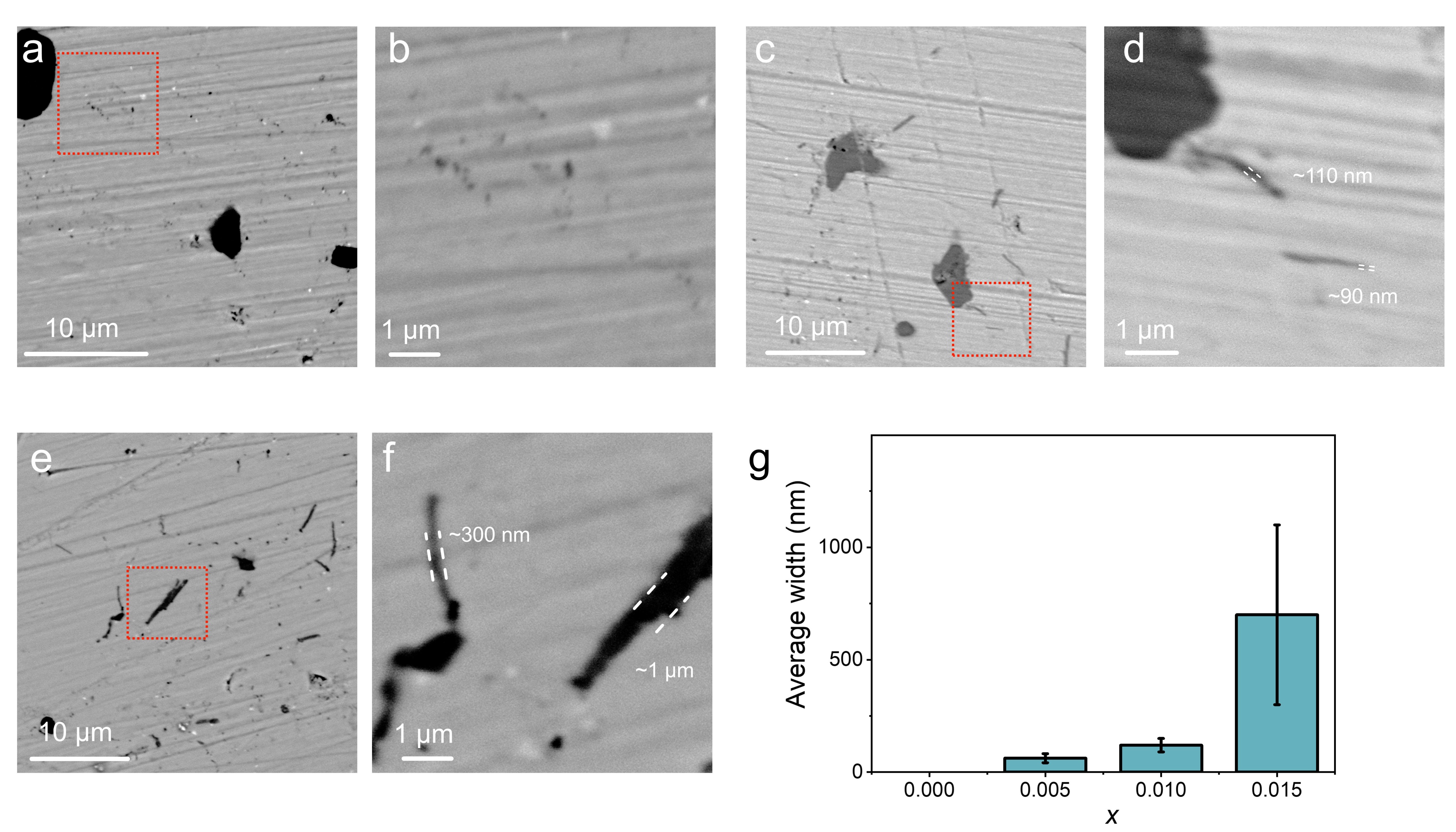


Figure S4. The average width of VSe_2_ precipitates. Scaning electron microscopy (SEM) images of (a) and (b) (Ge_0.82_Mn_0.04_Bi_0.04_Pb_0.1_Te)_0.995_(VSe_2_)_0.005_, (c) and (d) (Ge_0.82_Mn_0.04_Bi_0.04_Pb_0.1_Te)_0.99_(VSe_2_)_0.01_, (e) and (f) (Ge_0.82_Mn_0.04_Bi_0.04_Pb_0.1_Te)_0.985_(VSe_2_)_0.015_, (g) The average width of VSe_2_ precipitates of (Ge_0.82_Mn_0.04_Bi_0.04_Pb_0.1_Te)_1-x_(VSe_2_)_x_ (x=0.005-0.015).


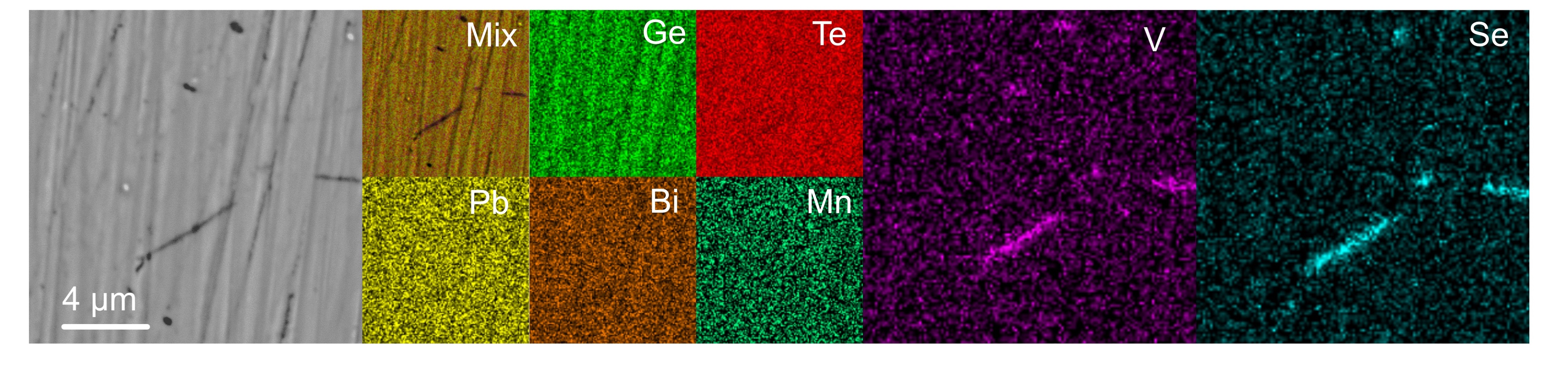


Figure S5. Element composition of VSe_2_ nano wire precipitates. Scaning electron microscopy (SEM) image with its energy dispersive (EDS) mappings of (Ge_0.82_Mn_0.04_Bi_0.04_Pb_0.1_Te)_0.99_(VSe_2_)_0.01_.


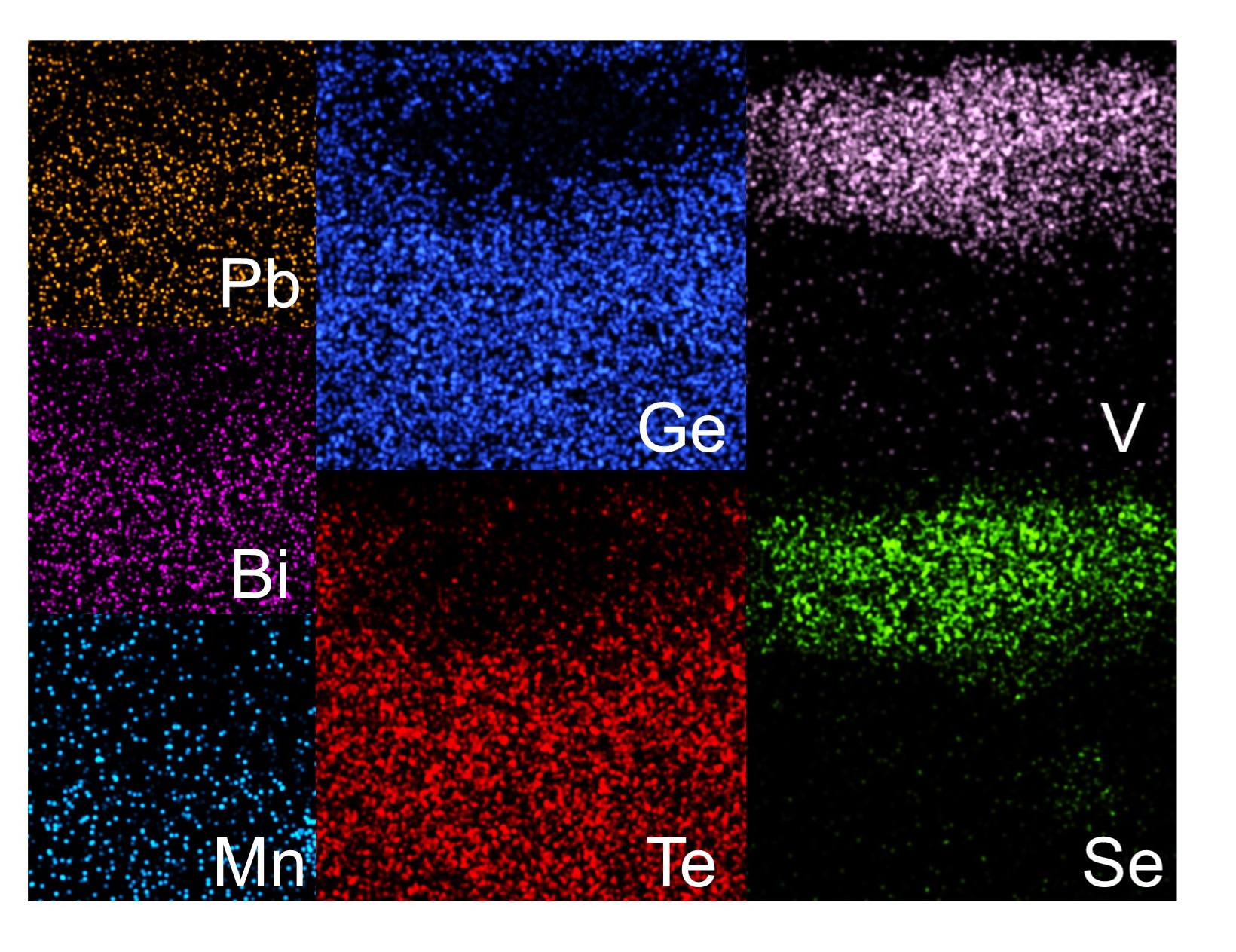


Figure S6. The corresponding EDS of Figure 2D


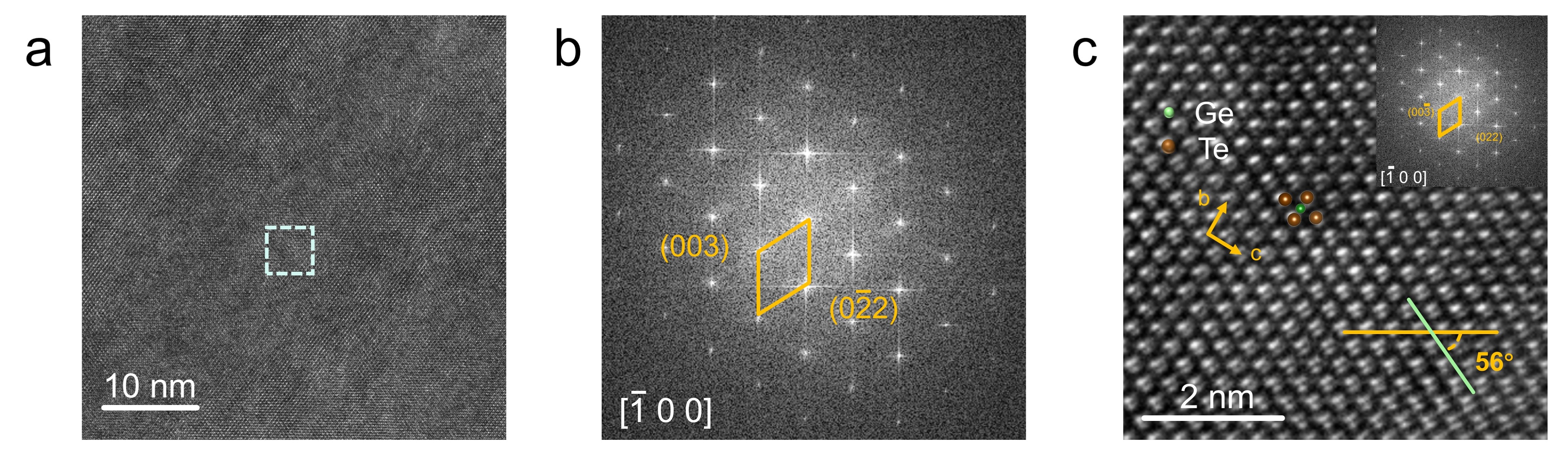


Figure S7. The microstructures of the Ge_0.82_Mn_0.04_Bi_0.04_Pb_0.1_Te matrix in (Ge_0.82_Mn_0.04_Bi_0.04_Pb_0.1_Te)_0.99_(VSe_2_)_0.01_ sample. (a) Transmission electron microscopy image of the Ge_0.82_Mn_0.04_Bi_0.04_Pb_0.1_Te matrix. (b) Fast Fourier Transform (FFT) pattern of the white-squared area of a (c) The high-resolution TEM (HRTEM) image of the white-squared area of a.


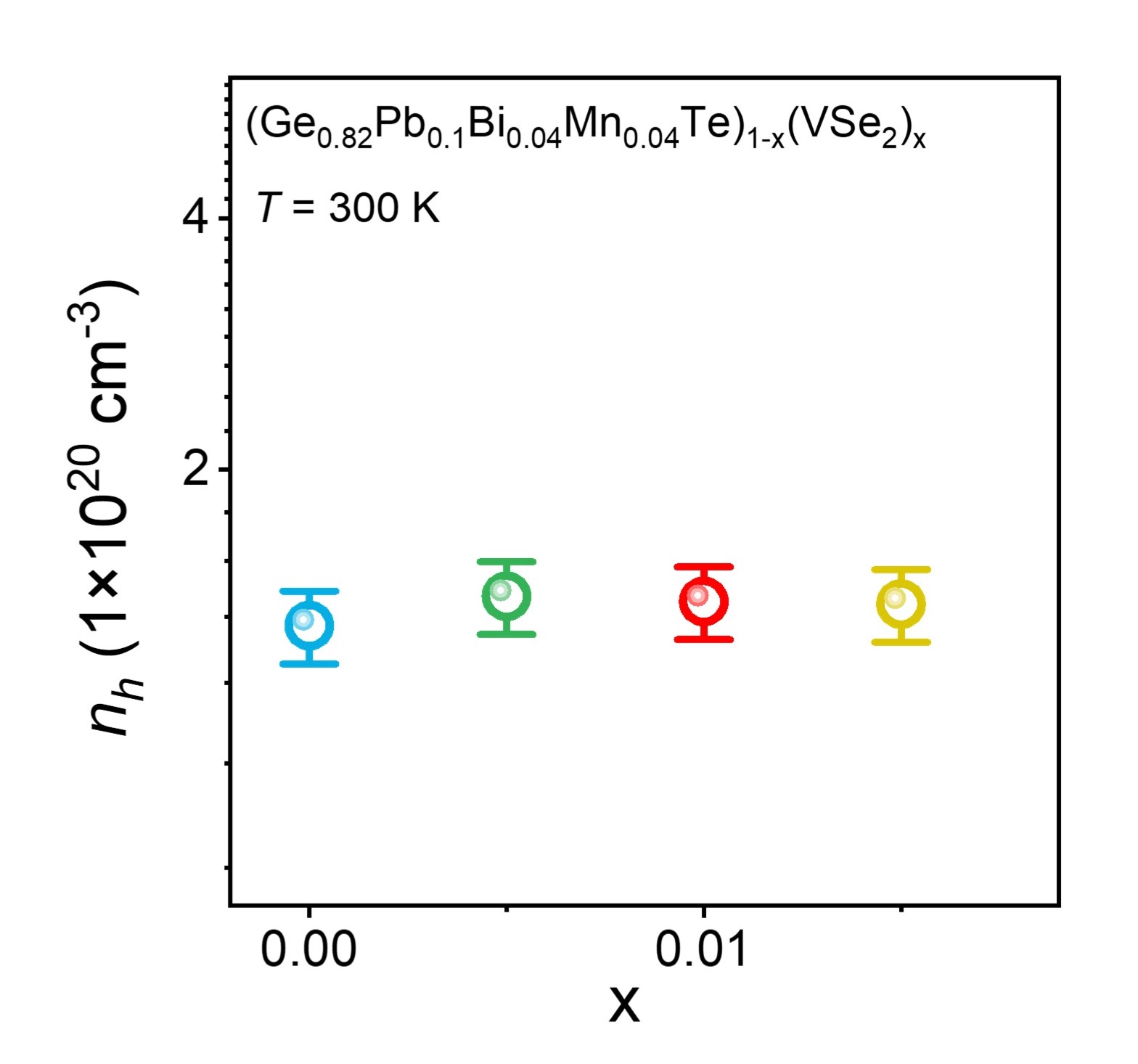


Figure S8. Influence of VSe_2_ nanowire precipitates on carrier concentration (*n*_h_). Composition-dependent *n*_h_ of the (Ge_0.82_Mn_0.04_Bi_0.04_Pb_0.1_Te)_1-x_(VSe_2_)_x_ (x=0-0.015) samples.


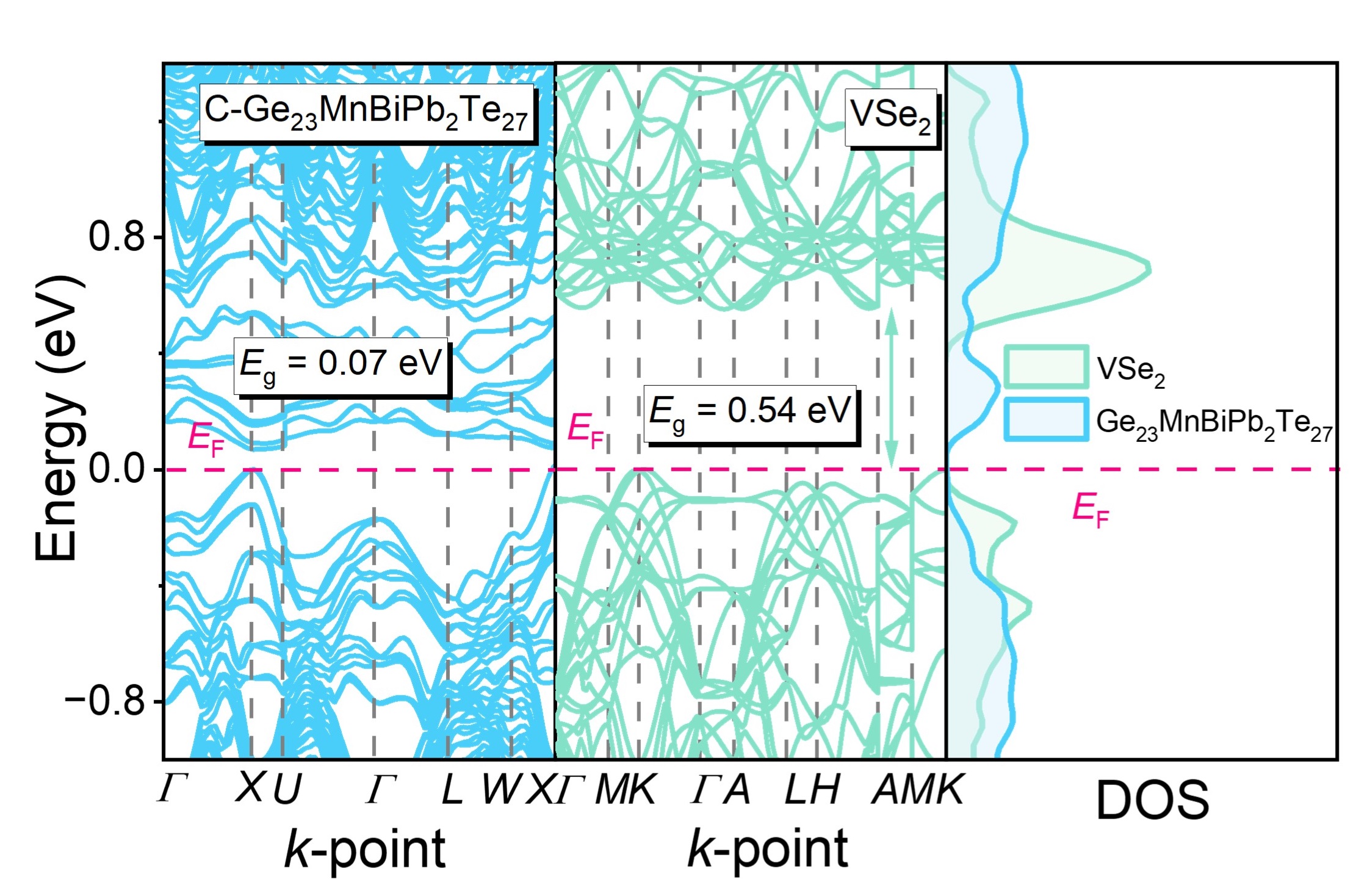


Figure S9. Band structures of C-GeTe. Band structures calculated for C-Ge_23_MnBiPb_2_Te_27_ and VSe_2_, with a comparison of the electronic density of states (DOS).


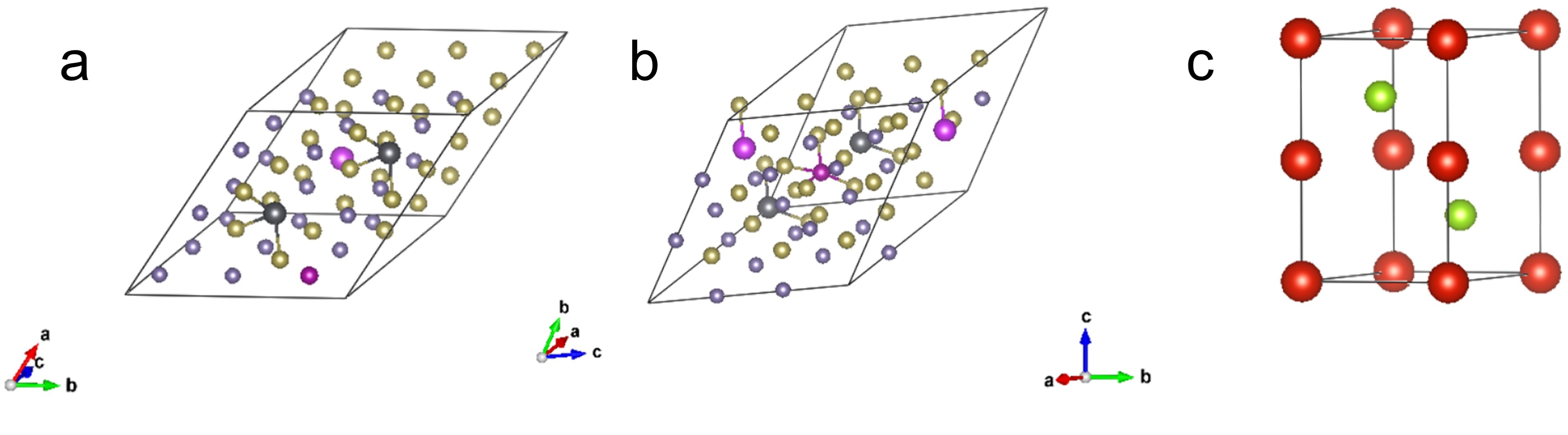


Figure S10. The atomic structure models of DFT calculations. (a)R-Ge23MnBiPb2Te27, (b) C-Ge23MnBiPb2Te27 and (c) VSe2.


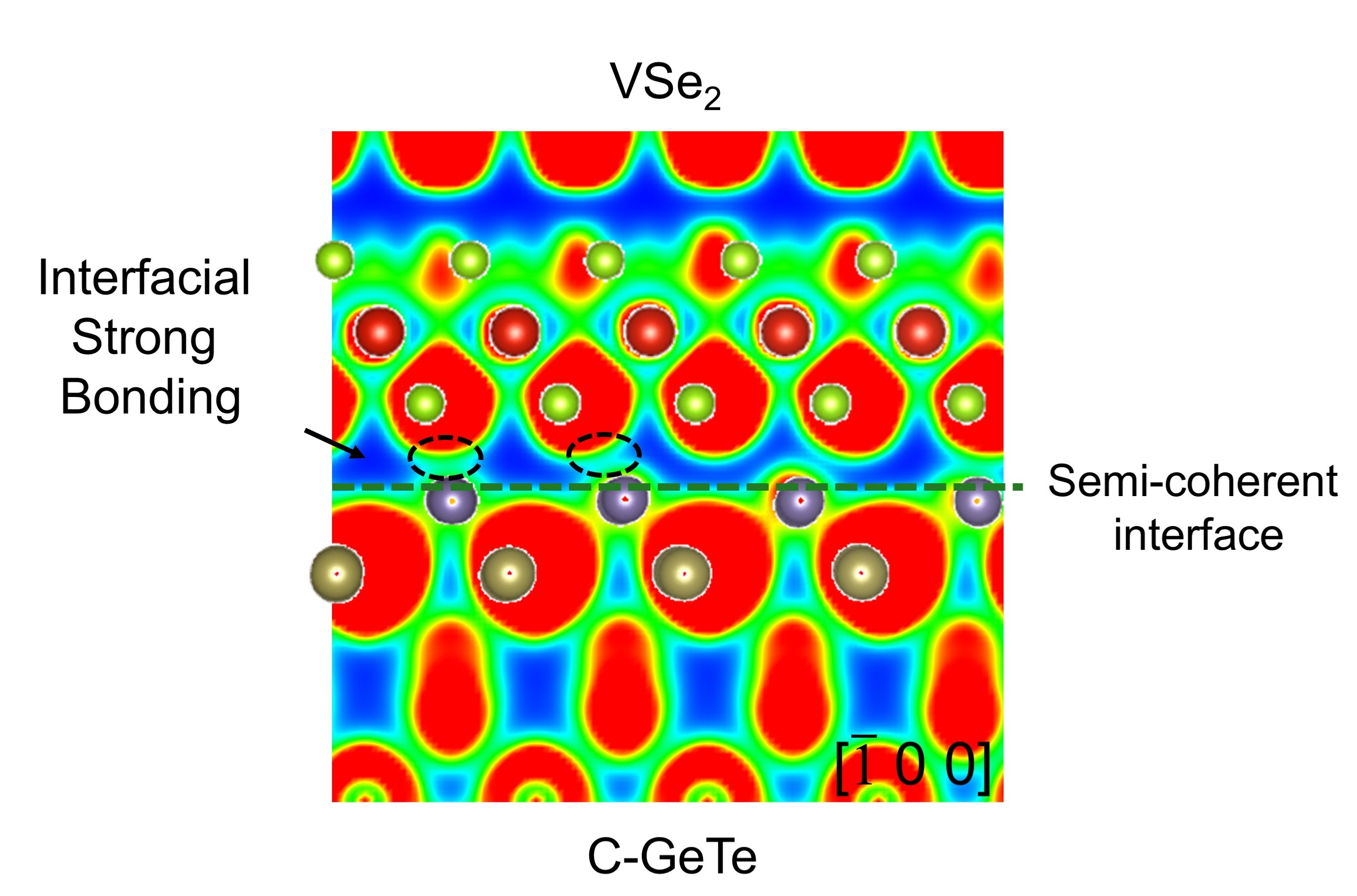


Figure S11. 2D mappings of electron localization function (ELF)for C-GeTe/VSe_2_ interfaces.


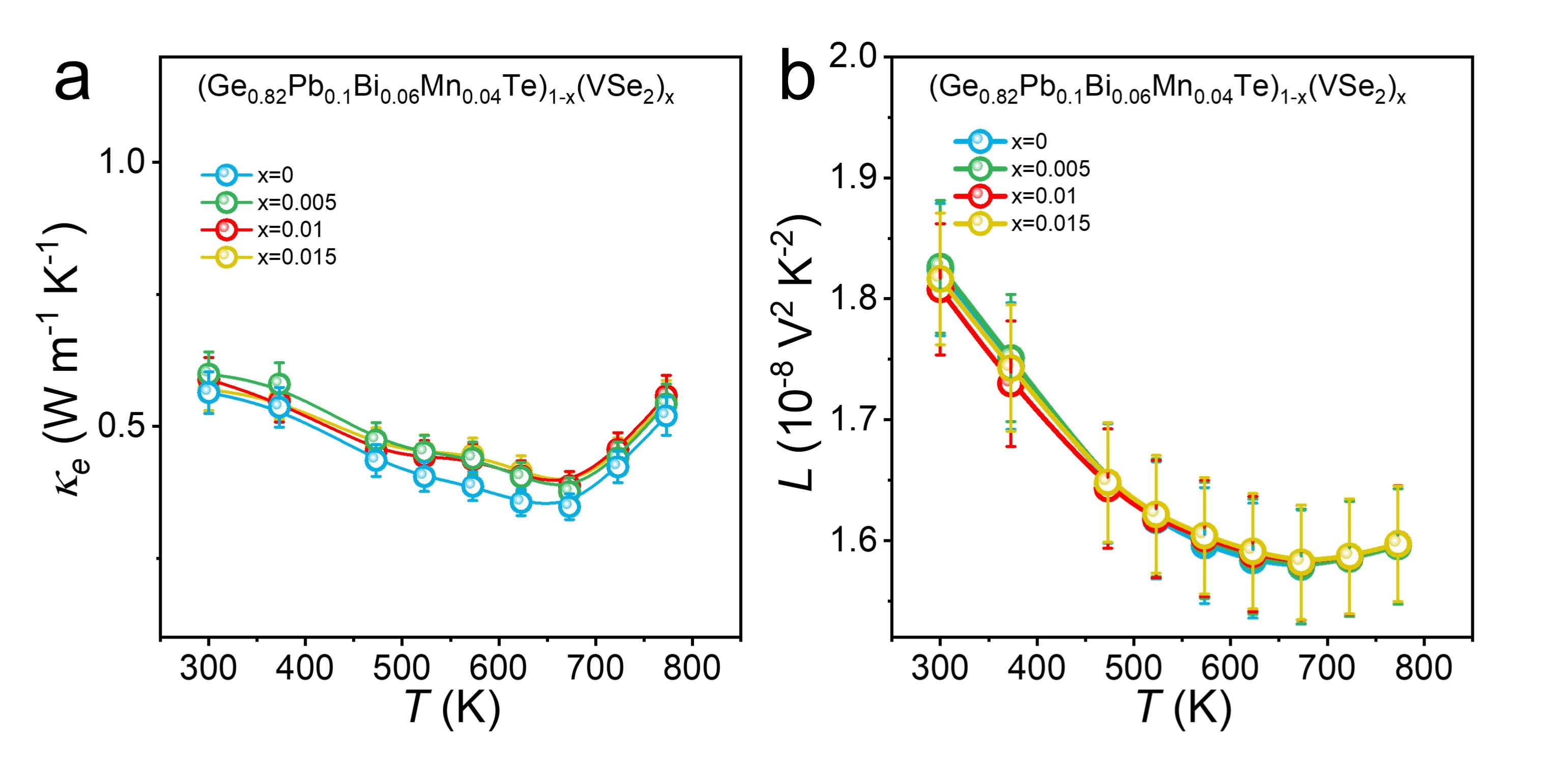


Figure S12. Electrical thermal conductivity (*κ*_e_) and Lorenz number (*L*) of (Ge_0.82_Mn_0.04_Bi_0.04_Pb_0.1_Te)_1-x_(VSe_2_)_x_ (x=0-0.015) samples. (a) Temperature-dependent *κ*_e_ in the (Ge_0.82_Mn_0.04_Bi_0.04_Pb_0.1_Te)_1-x_(VSe_2_)_x_ (x=0-0.015) samples. (b) Temperature-dependent *L* in the (Ge_0.82_Mn_0.04_Bi_0.04_Pb_0.1_Te)_1-x_(VSe_2_)_x_ (x=0-0.015) samples.


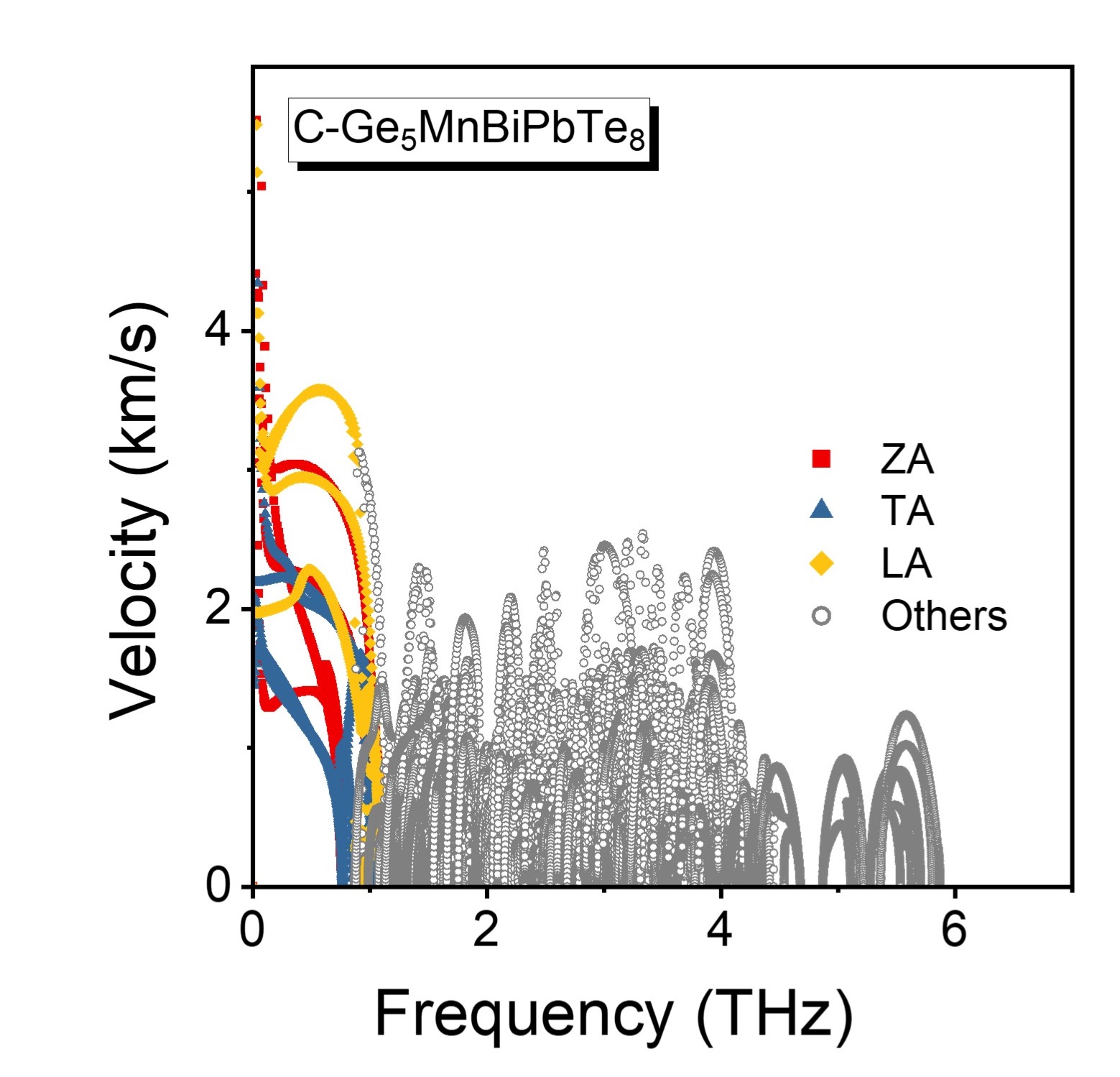


Figure S13. Phonon group velocity (*v*_g_) by DFT calculation of C-Ge_5_MnBiPbTe_8_.


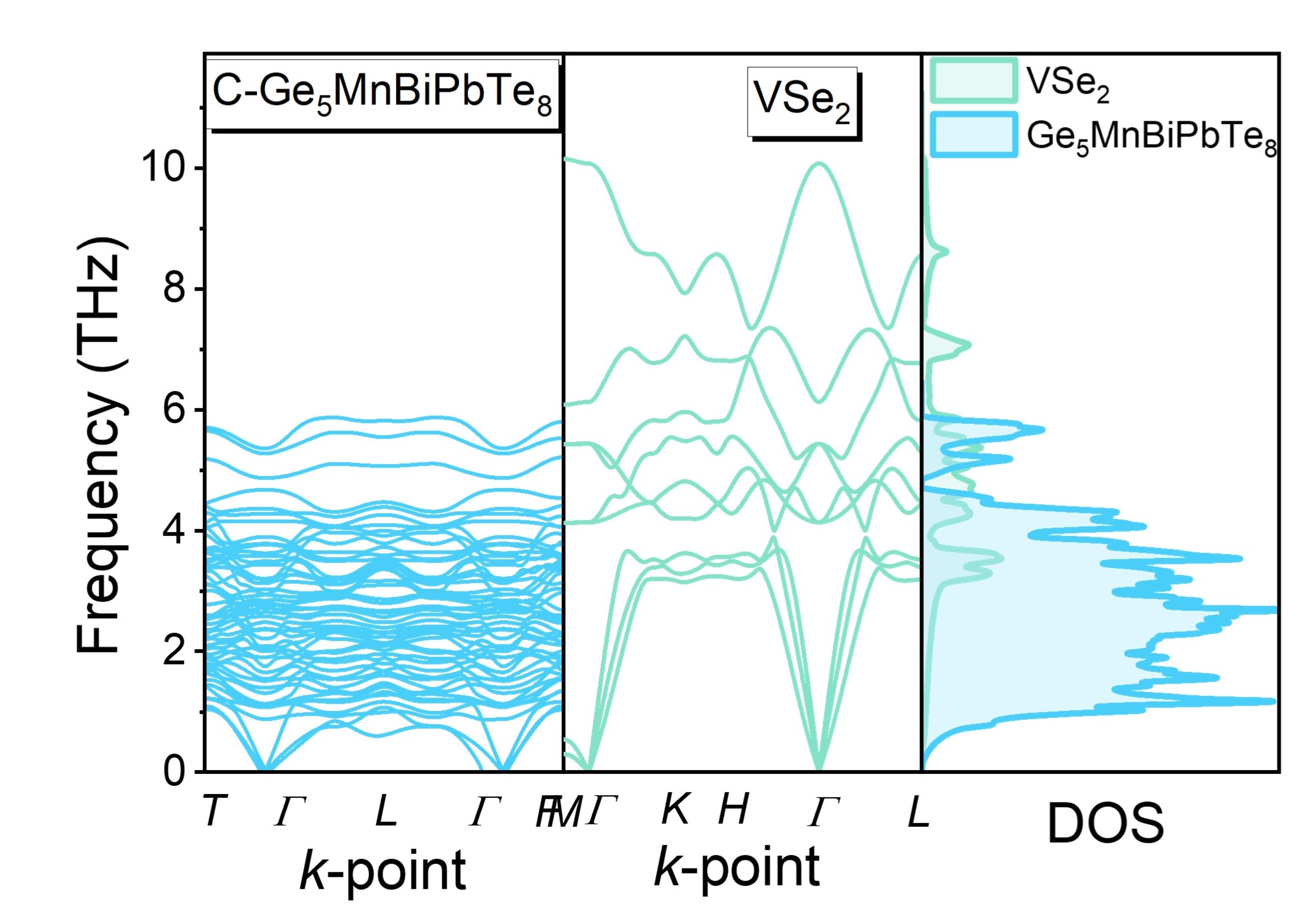


Figure S14. Phonon spectra of C-GeTe. Phonon spectra of C-Ge_5_MnBiPbTe_8_ and VSe_2_, along with a comparison of their phonon DOS.


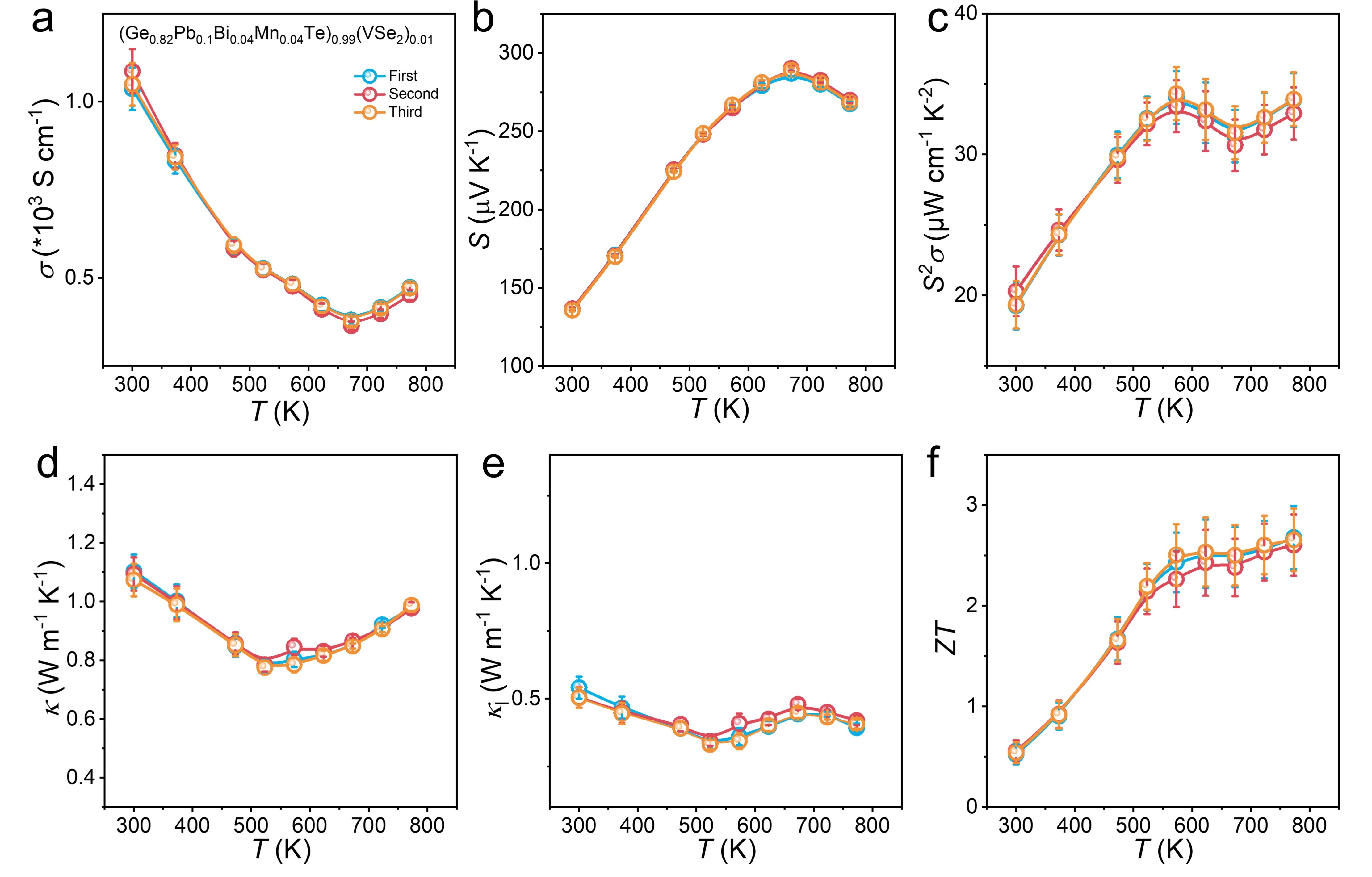


Figure S15. Repeatability of thermoelectric measurements for (Ge_0.82_Mn_0.04_Bi_0.04_Pb_0.1_Te)_0.99_(VSe_2_)_0.01_ pellet. Temperature-dependent (a) electrical conductivity *σ*, (b) *S*, (c) power factor *S^2^σ*, (d) total thermal conductivity *κ*, (e) lattice thermal conductivity *κ*_l_ and (f) *ZT*.


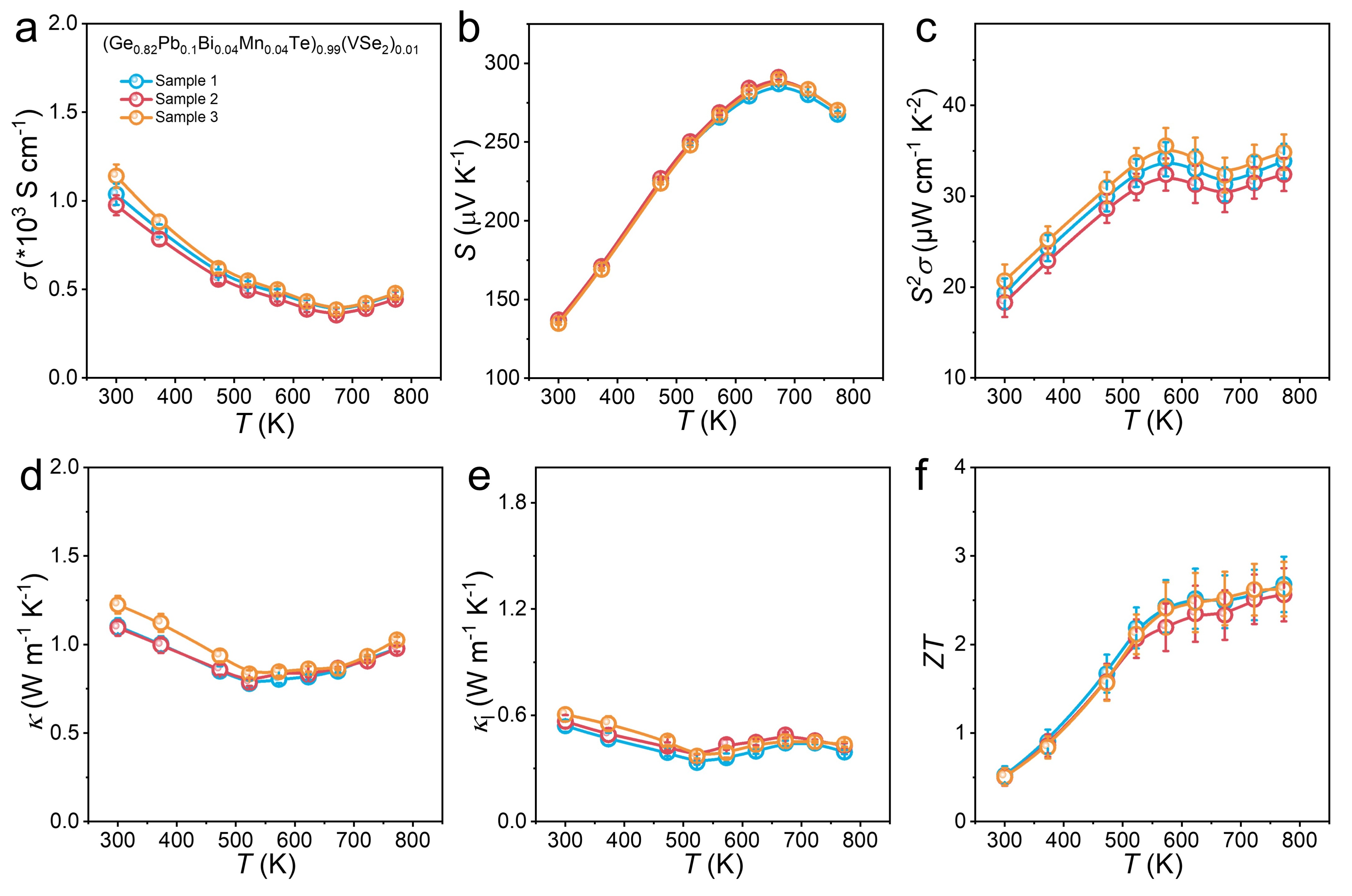


Figure S16. Reproducibility of thermoelectric measurements for (Ge_0.82_Mn_0.04_Bi_0.04_Pb_0.1_Te)_0.99_(VSe_2_)_0.01_ pellet. Temperature-dependent (a) *σ*, (b) *S*, (c)*S^2^σ*, (d) *κ*, (e) *κ*_l_ and (f) *ZT*.


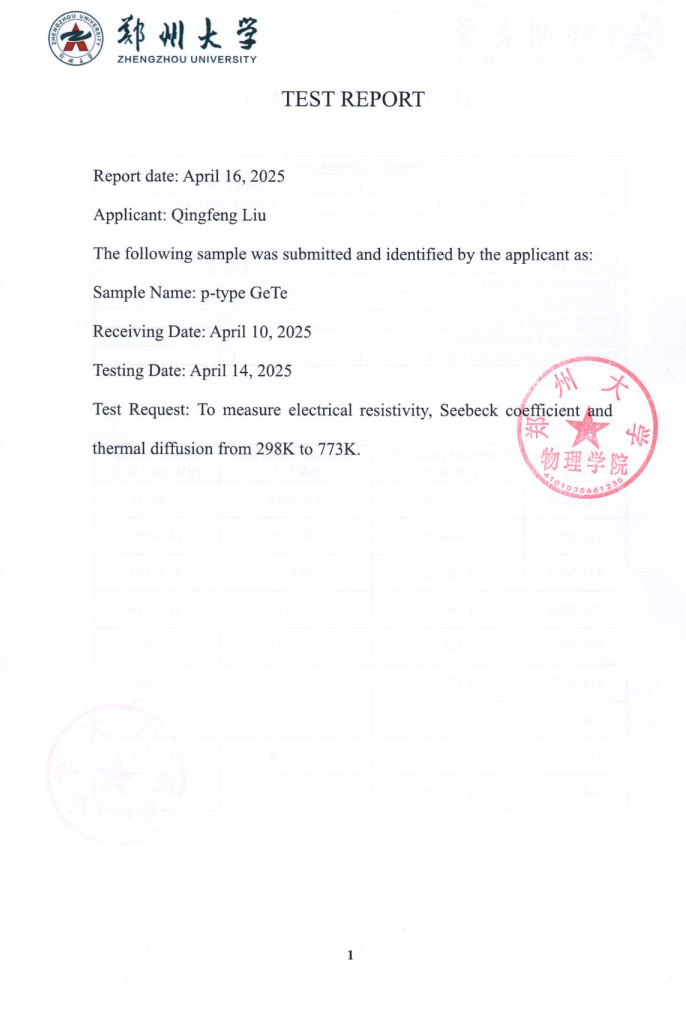

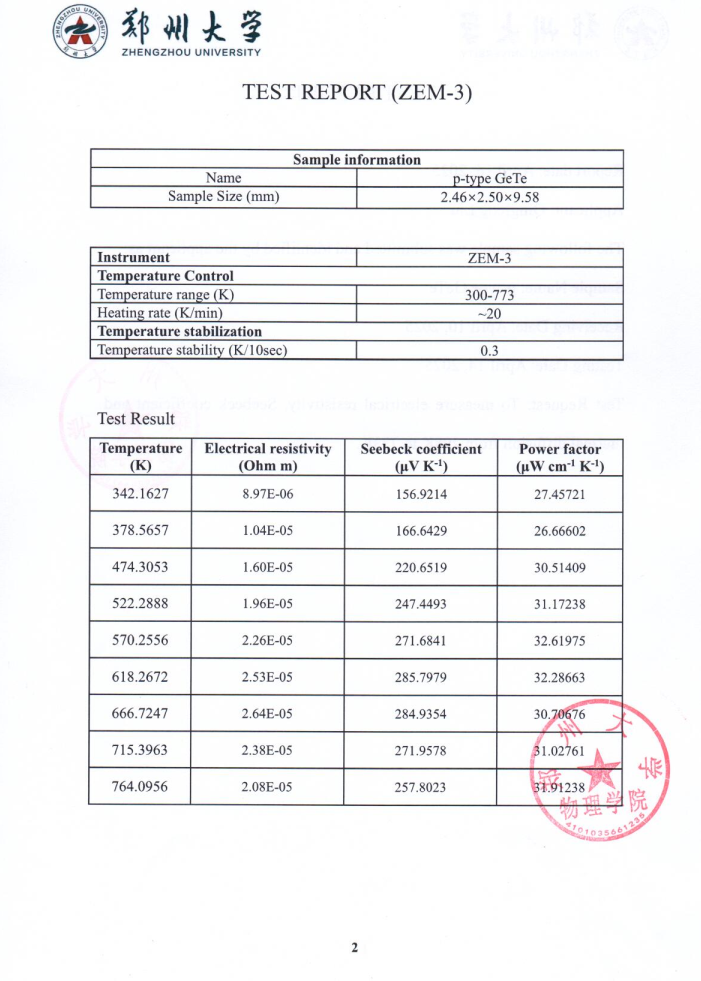

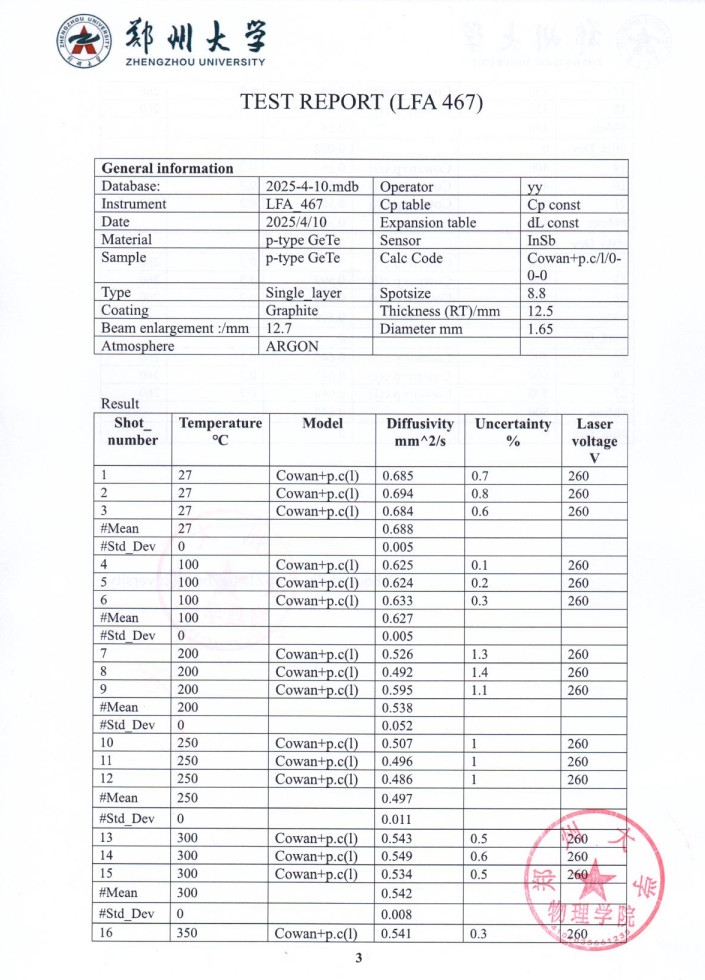

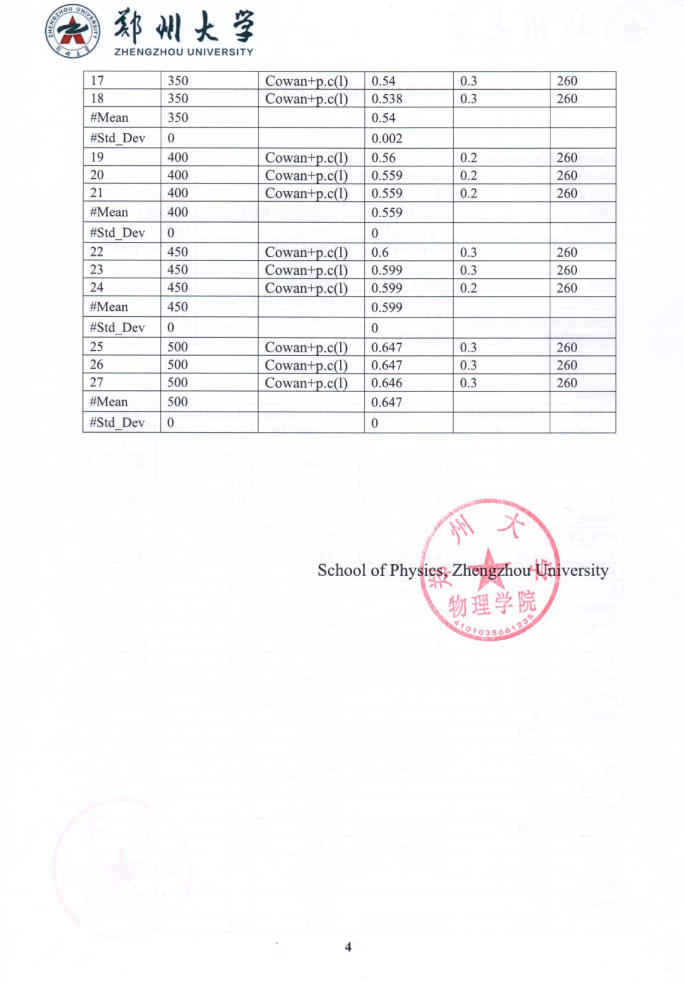

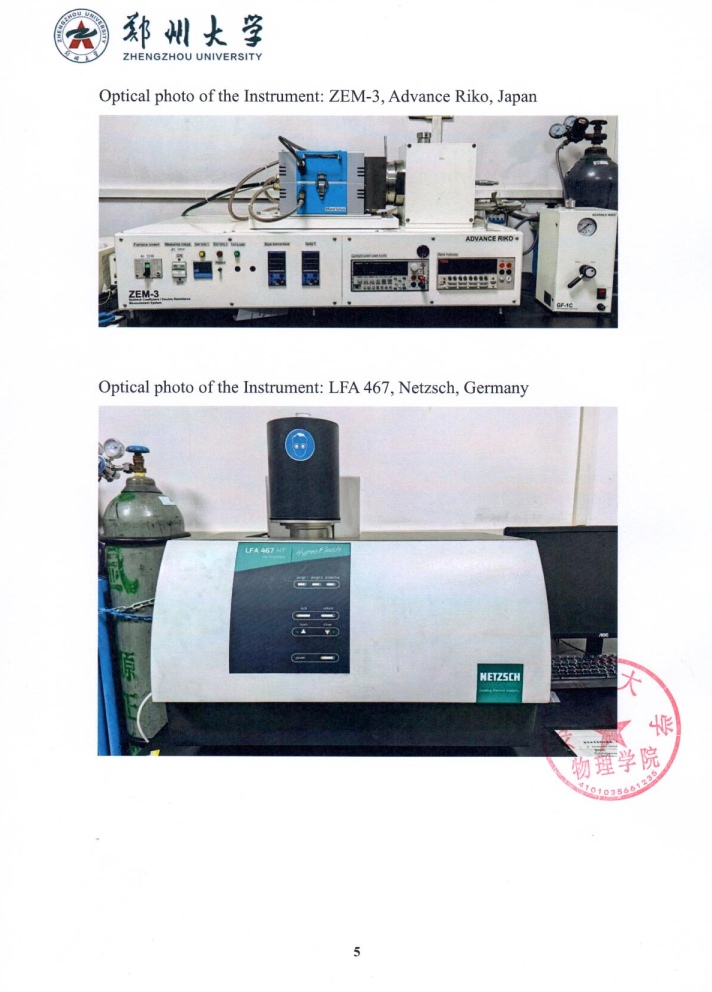


Figure S17. Test report from third-party measurement.


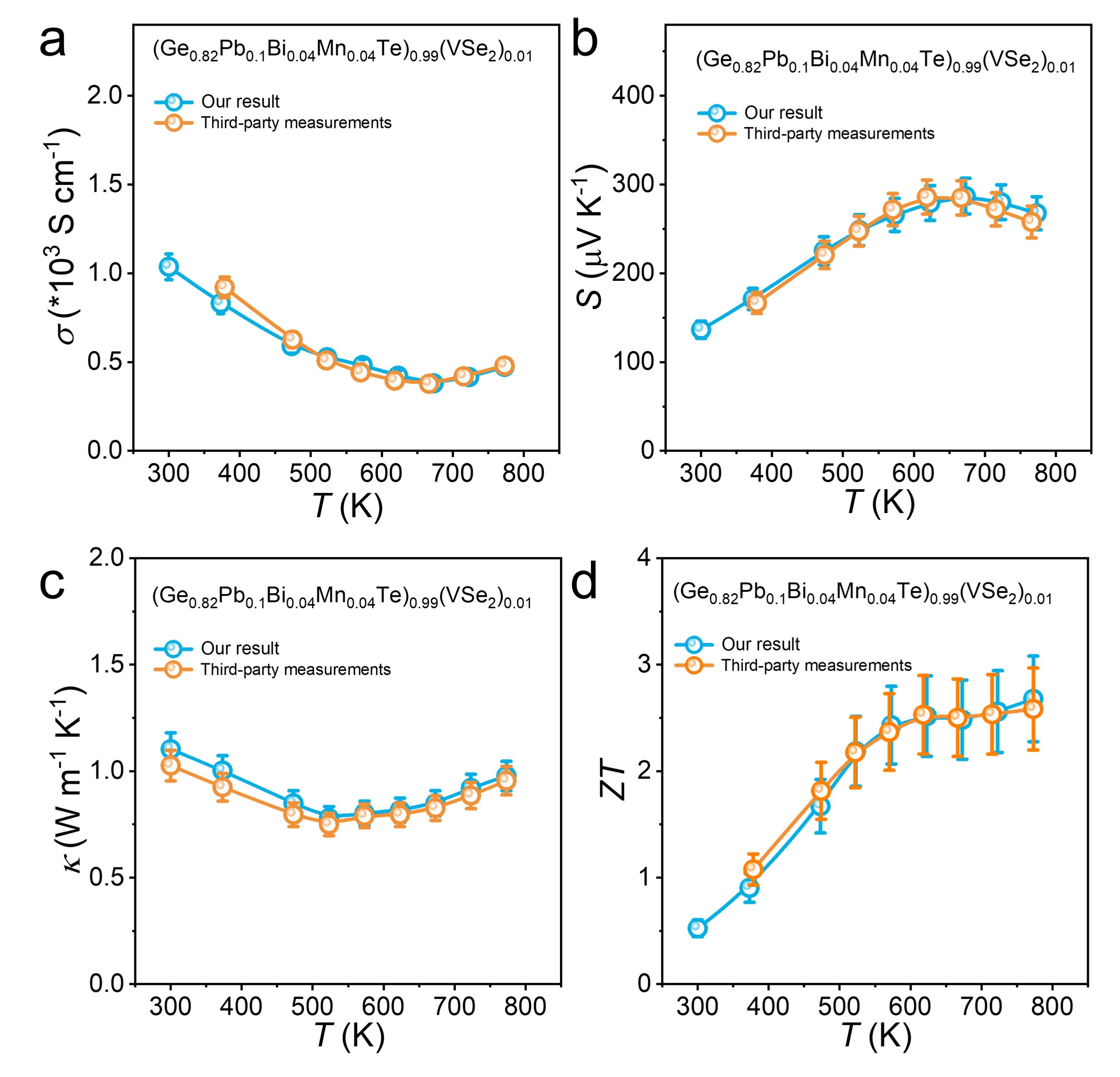


Figure S18. The thermoelectric performance of third-party measurement of (Ge_0.82_Mn_0.04_Bi_0.04_Pb_0.1_Te)_0.99_(VSe_2_)_0.01_ pellet and compared it with our results. Temperature-dependent (a) *σ*, (b) *S*, (c)*S^2^σ*, (d) *κ*, (e) *κ*_l_ and (f) *ZT*.


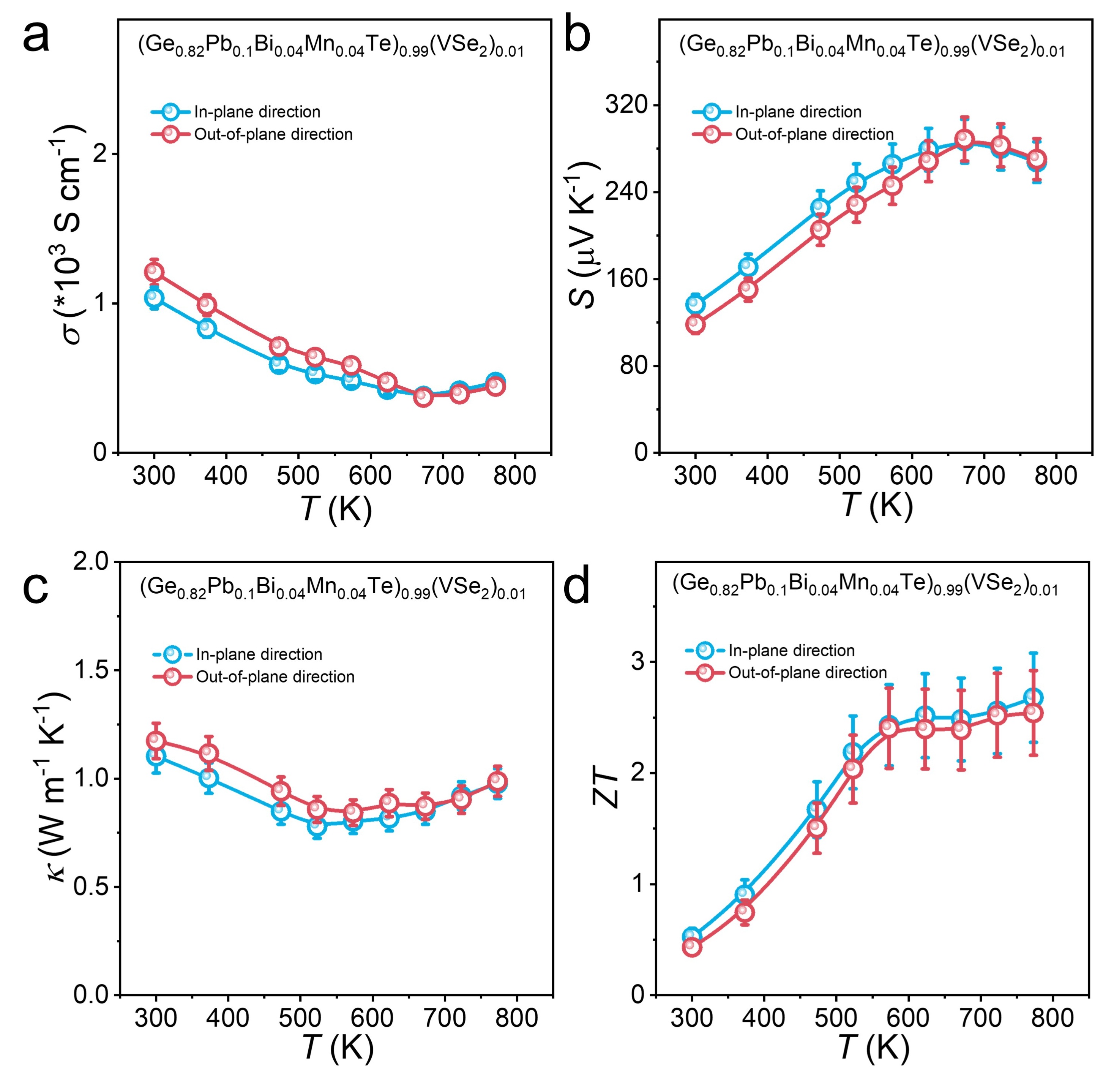


Figure S19. Thermoelectric measurement of different pressure directions for (Ge_0.82_Mn_0.04_Bi_0.04_Pb_0.1_Te)_0.99_(VSe_2_)_0.01_ pellet. Temperature-dependent (a) *σ*, (b) *S*, (c)*κ* and (d) *ZT*.


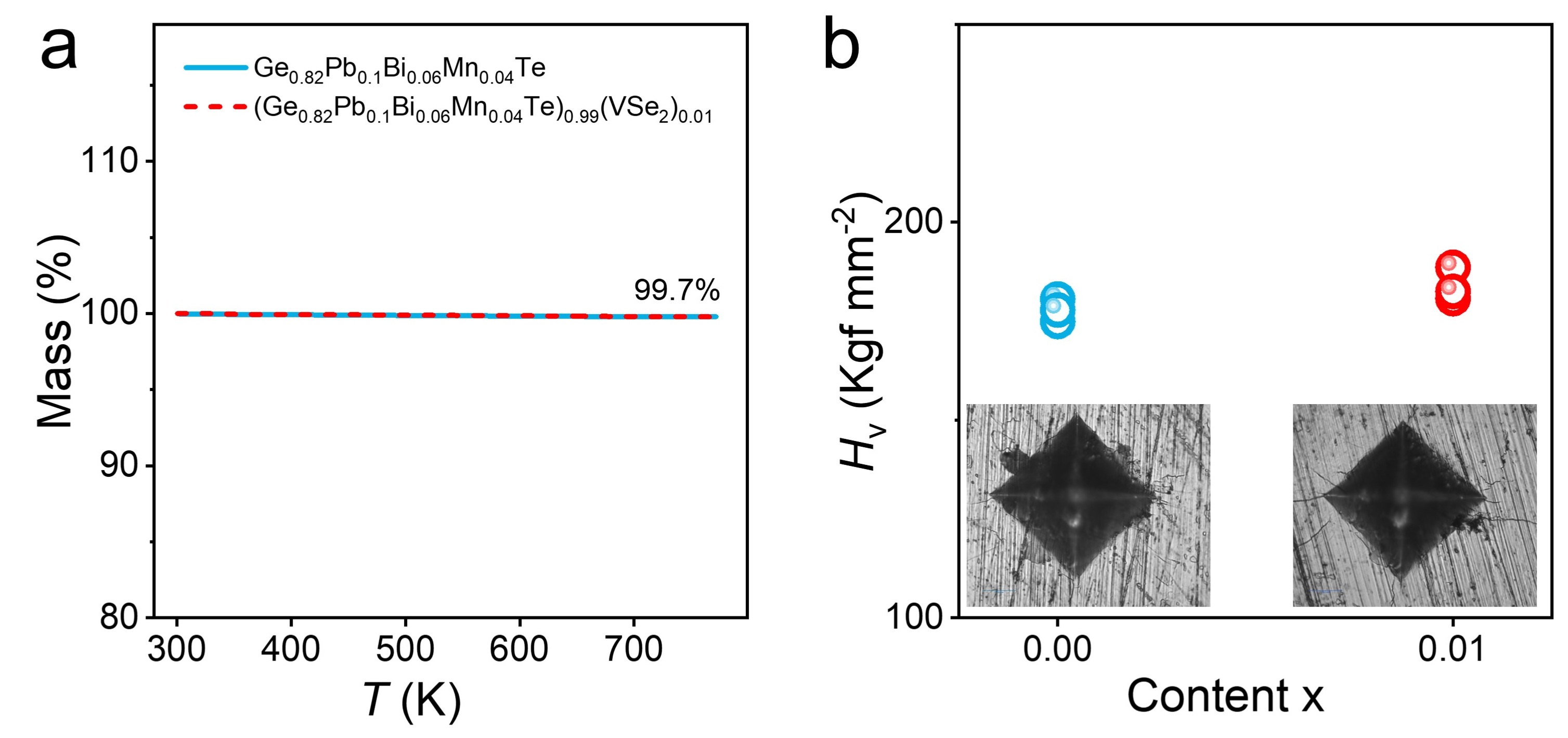


Figure S20. The influence of introducing VSe_2_ on thermal stability and mechanical properties. (a) Thermogravimetric Analysis (TGA) curves of Ge_0.82_Mn_0.04_Bi_0.04_Pb_0.1_Te and (Ge_0.82_Mn_0.04_Bi_0.04_Pb_0.1_Te)_0.99_(VSe_2_)_0.01_, (b) Vickers hardness of Ge_0.82_Mn_0.04_Bi_0.04_Pb_0.1_Te and (Ge_0.82_Mn_0.04_Bi_0.04_Pb_0.1_Te)_0.99_(VSe_2_)_0.01_ and corresponding optical micrographs of the indentations.


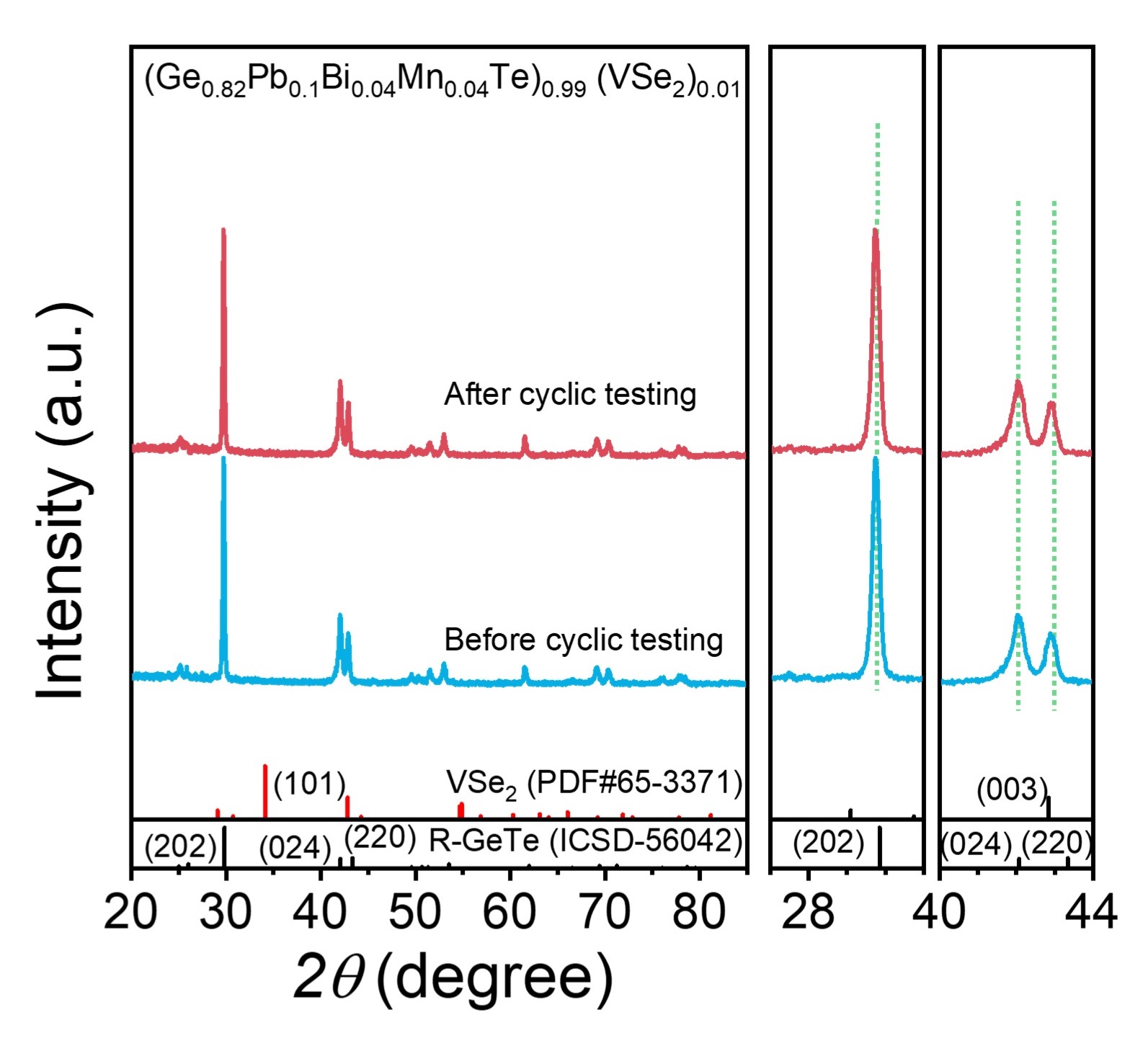


Figure S21. XRD analysis on (Ge_0.82_Mn_0.04_Bi_0.04_Pb_0.1_Te)_0.99_(VSe_2_)_0.01_ sample before and after the thermal cycling test.


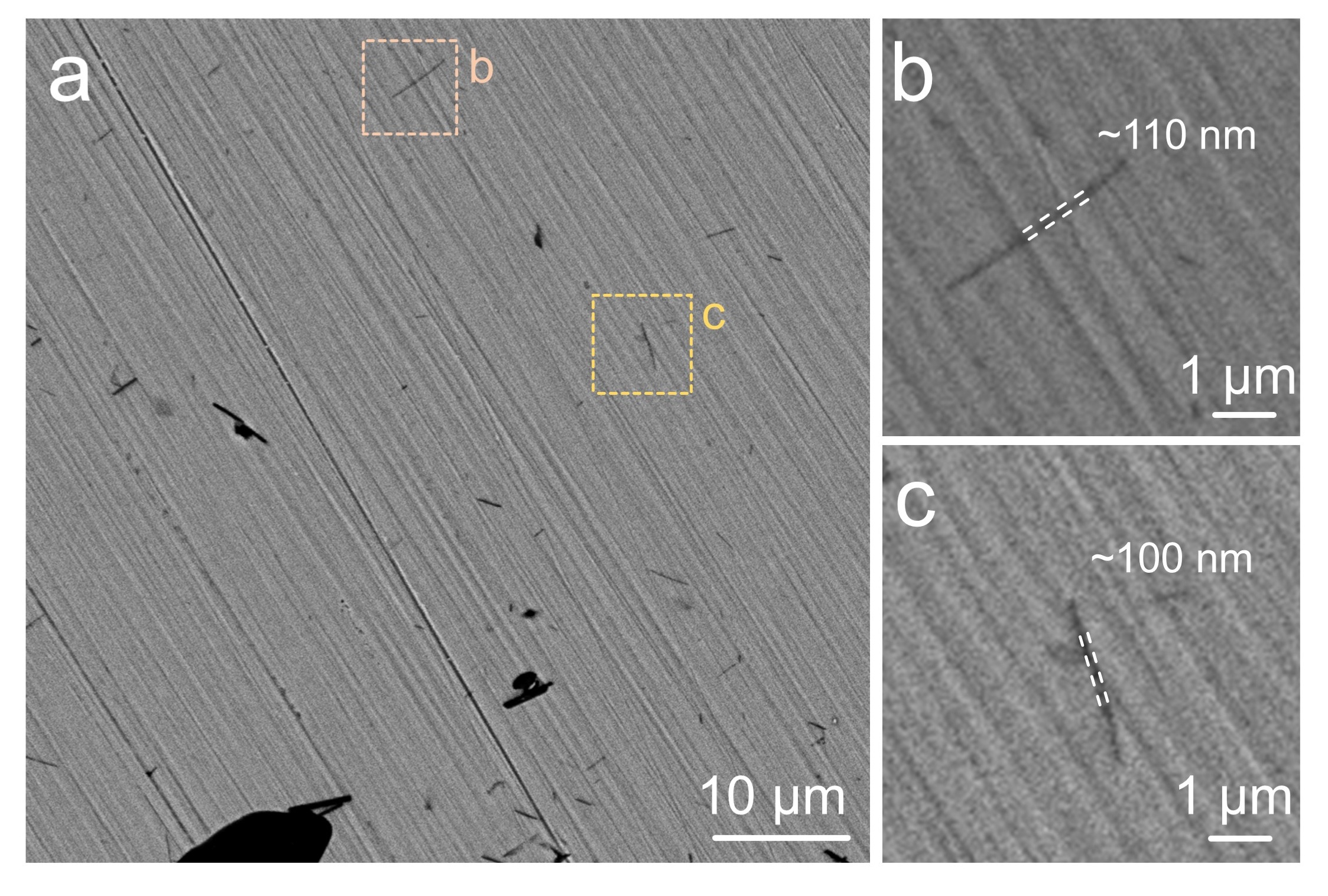


Figure S22. SEM analysis on the (Ge_0.82_Mn_0.04_Bi_0.04_Pb_0.1_Te)_0.99_(VSe_2_)_0.01_ sample after the thermal cycling test.


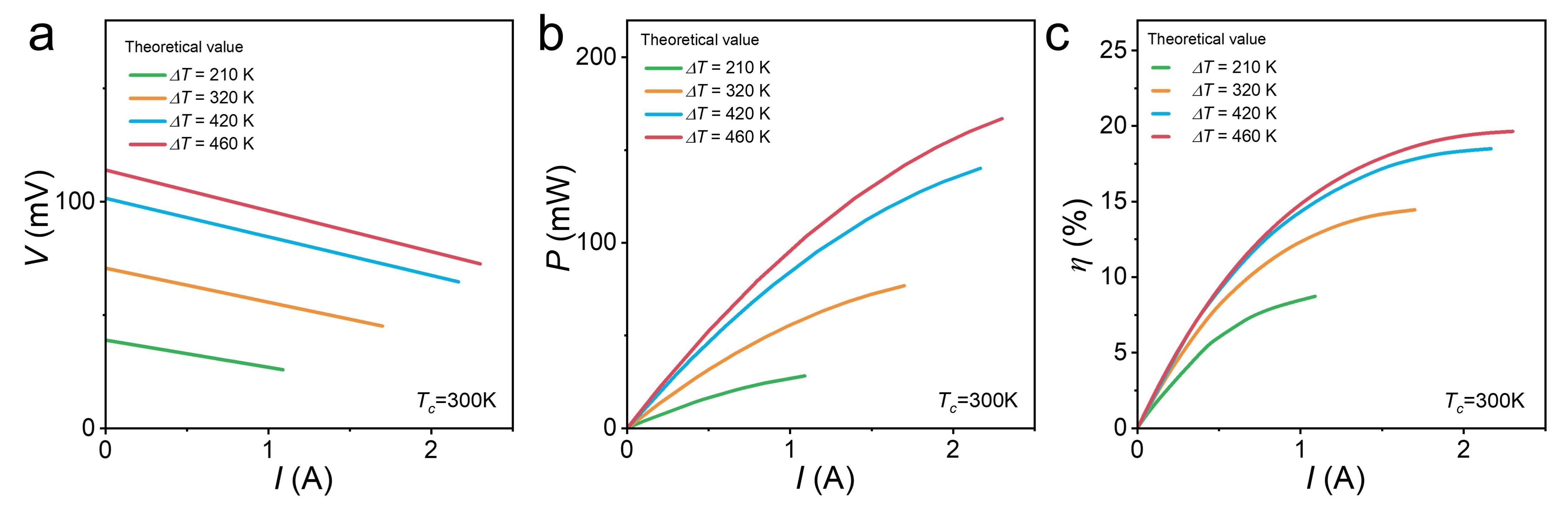


Figure S23. Simulation results of the (Ge_0.82_Mn_0.04_Bi_0.04_Pb_0.1_Te)_0.99_(VSe_2_)_0.01_ single leg device. Current (*I*)-dependent (a) Voltage *V* and (b) output power *P* (c) conversion efficiency *η*.


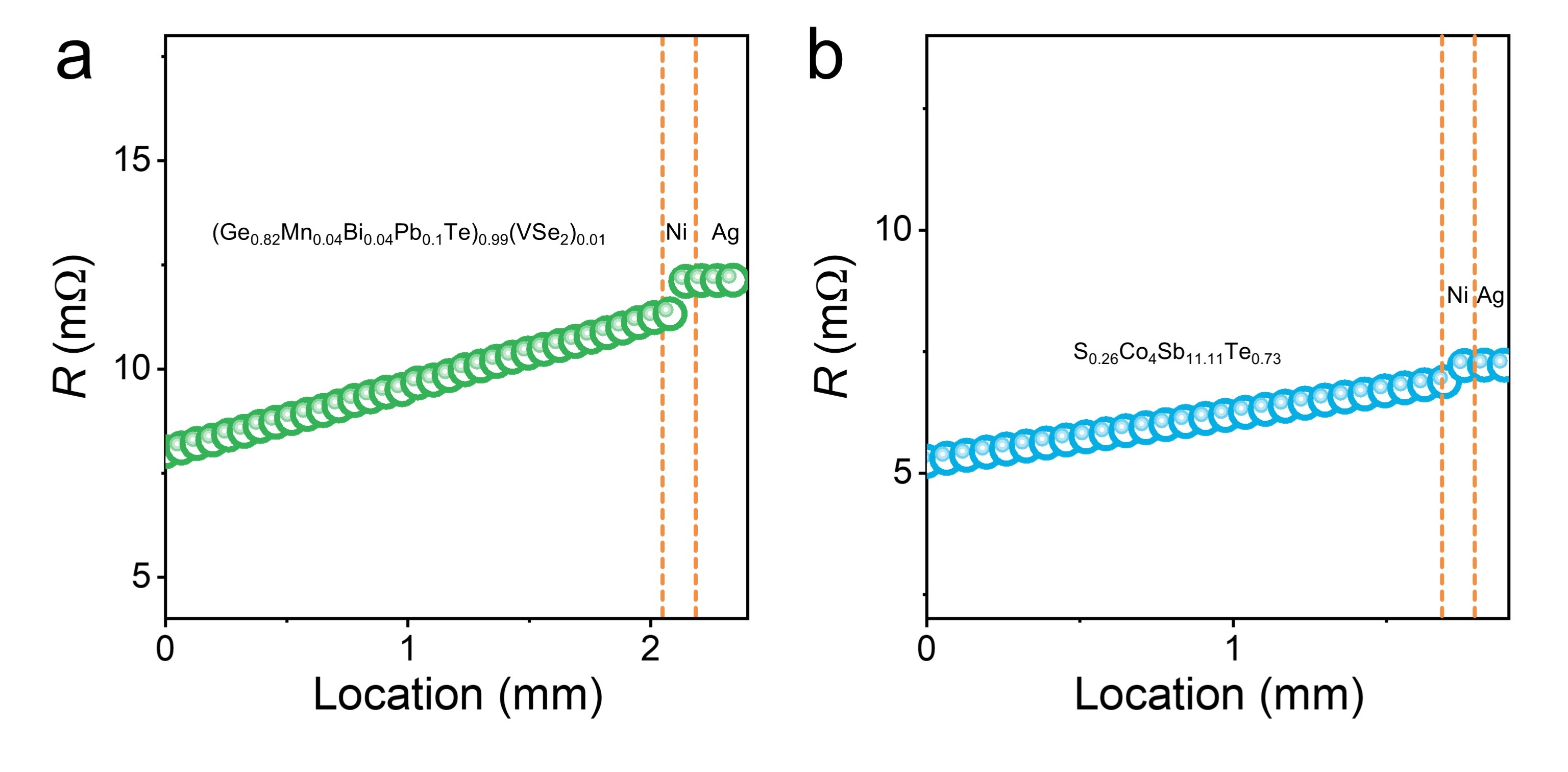


Figure S24. Resistance line scanning across the interfaces of the (a) (Ge_0.82_Mn_0.04_Bi_0.04_Pb_0.1_Te)_0.99_(VSe_2_)_0.01_/Ni/Ag leg, and (b) S_0.26_Co_4_Sb_11.11_Te_0.73_/Ni/Ag leg.


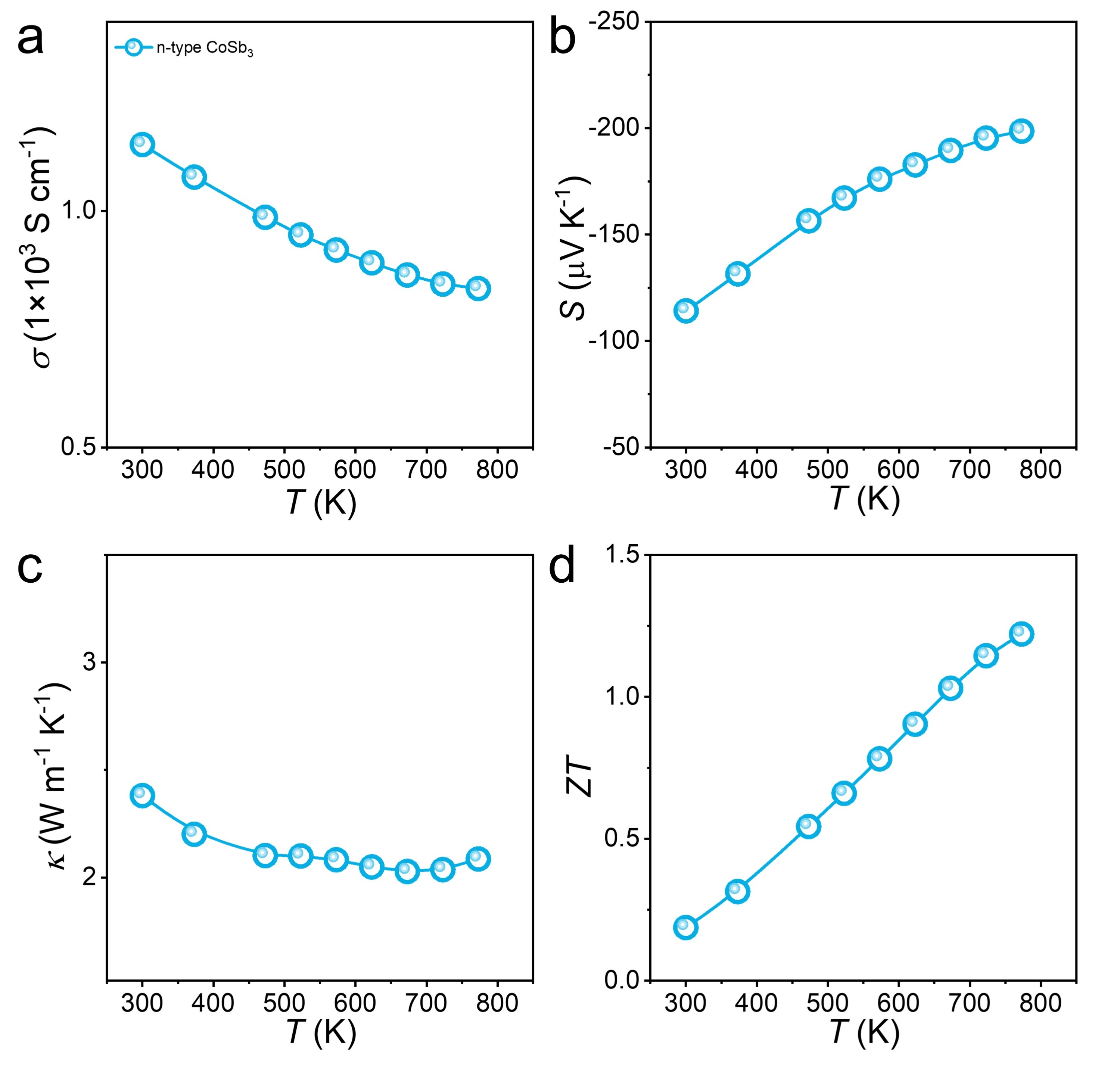


Figure S25. Thermoelectric performance of n-type S_0.26_Co_4_Sb_11.11_Te_0.73_. Temperature-dependent (a) *σ*, (b) *S*, (c) *κ*, and (d) *ZT*.


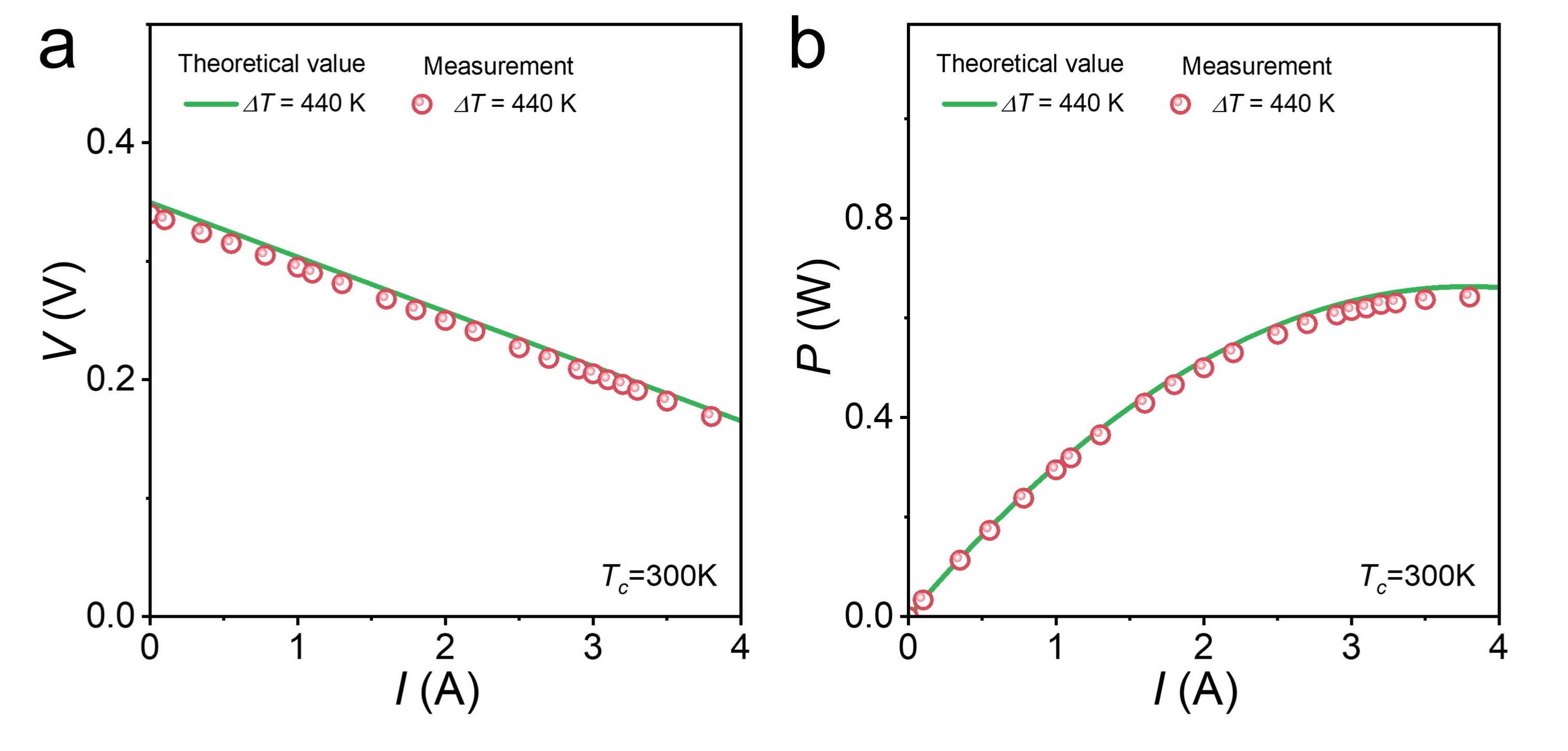


Figure S26. Comparison of simulated data (accounting for contact resistance) and actual data for the device under a *ΔT* of 440K. Current (*I*)-dependent (a) Voltage *V* and (b) output power *P*.


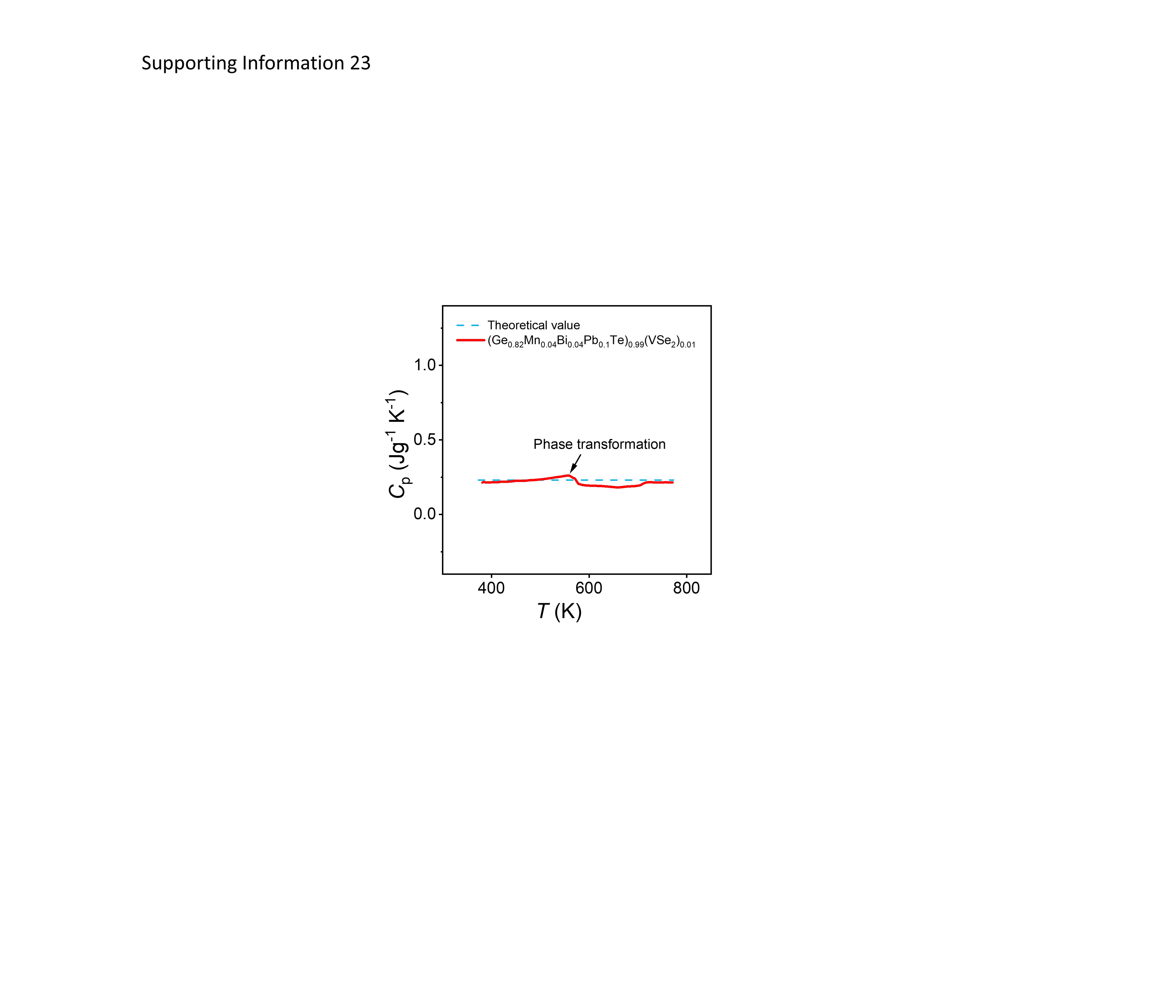


**Figure S27.** Measured heat capacity at constant pressure of (Ge_0.82_Mn_0.04_Bi_0.04_Pb_0.1_Te)_0.99_(VSe_2_)_0.01_.


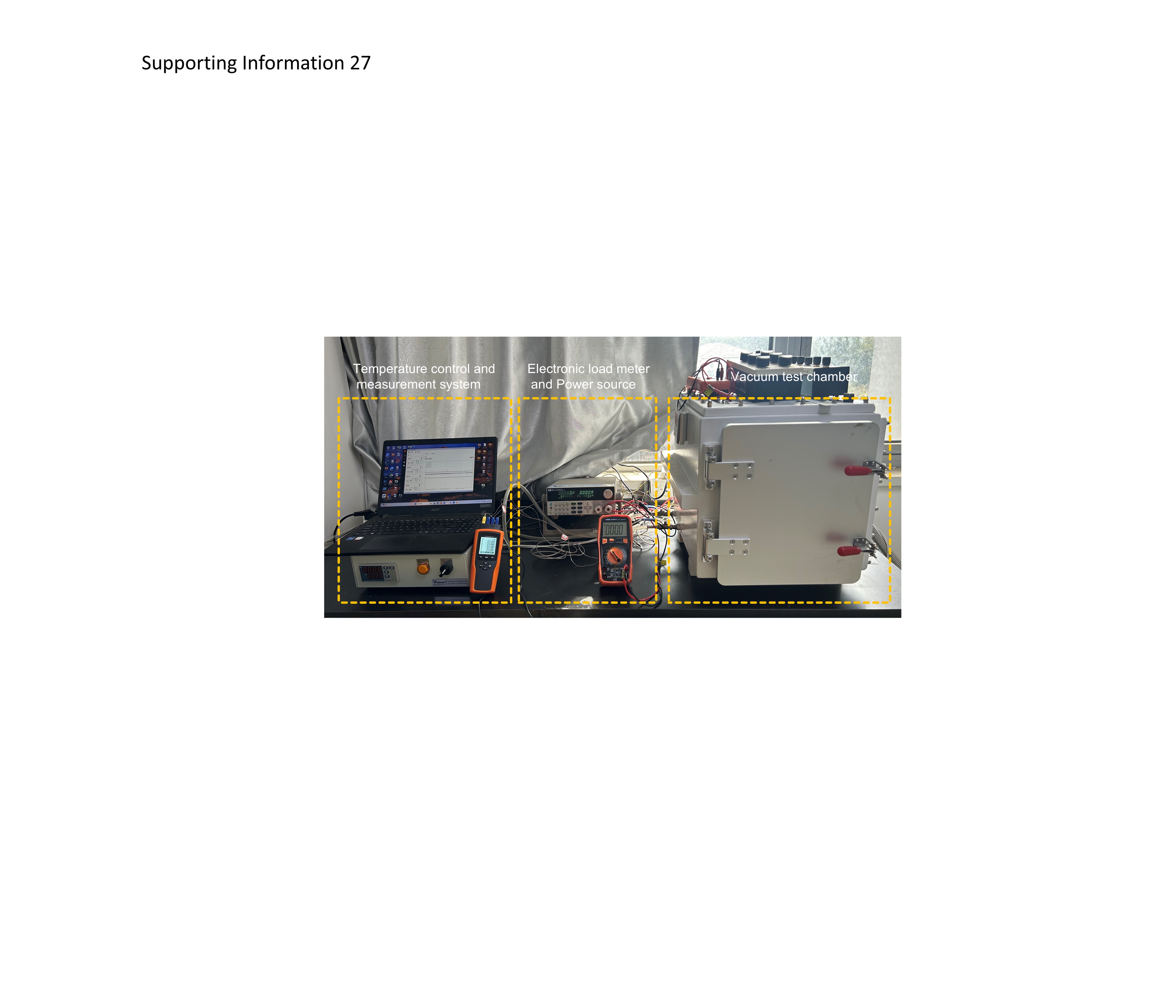


Figure S28. The home-made testing system for thermoelectric conversion efficiency (*η*) measurement.


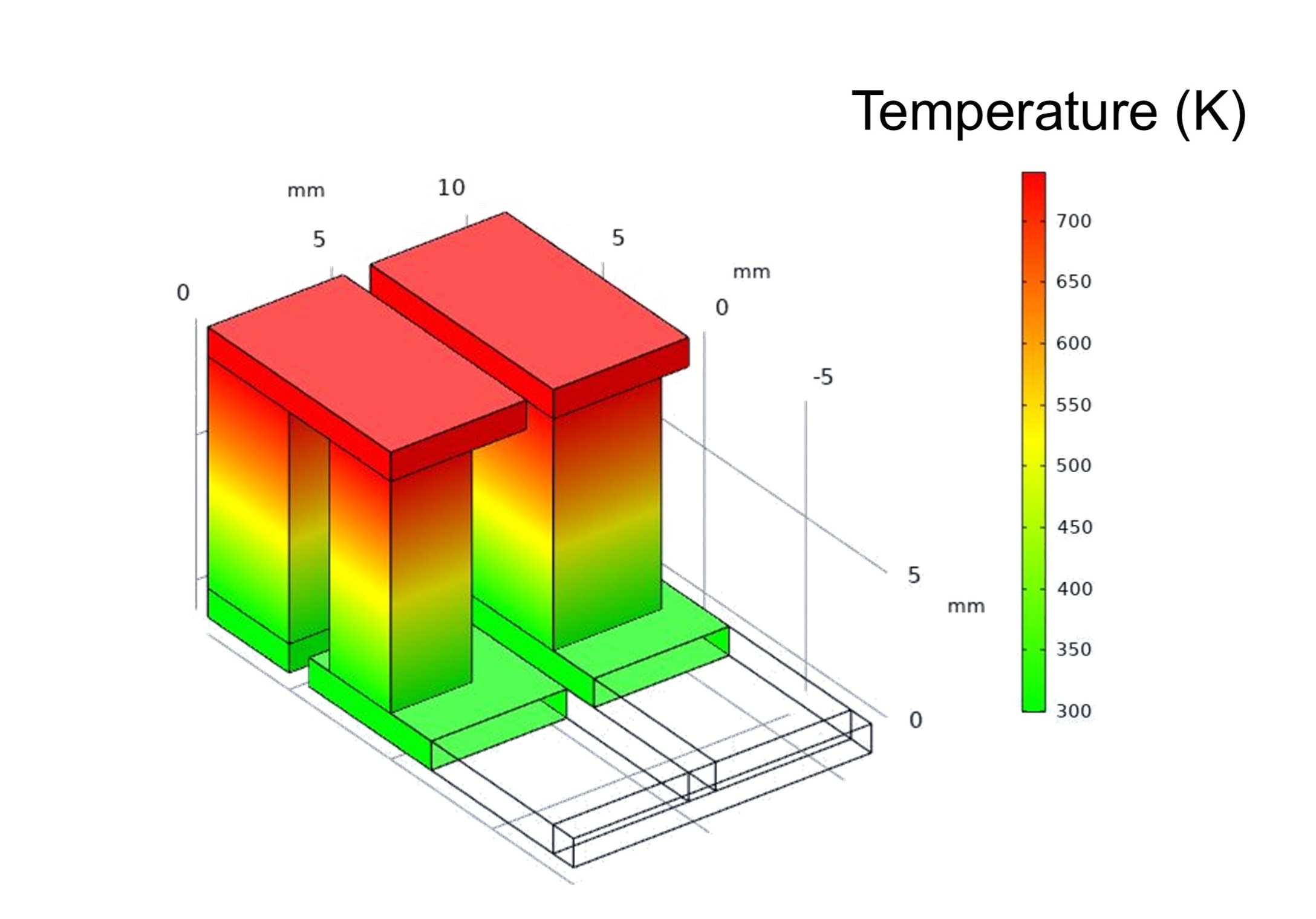


Figure S29. The model of module performance simulation.

Table S1. Value for material densities.

| Sample | Density (g cm^-3^) |
| --- | --- |
| Ge_0.82_Mn_0.04_Bi_0.04_Pb_0.1_Te | 6.45 |
| (Ge_0.82_Mn_0.04_Bi_0.04_Pb_0.1_Te)_0.995_(VSe_2_)_0.005_ | 6.42 |
| (Ge_0.82_Mn_0.04_Bi_0.04_Pb_0.1_Te)_0.99_(VSe_2_)_0.01_ | 6.40 |
| (Ge_0.82_Mn_0.04_Bi_0.04_Pb_0.1_Te)_0.985_(VSe_2_)_0.015_ | 6.39 |

Table S2. Error Bar Determination

| T(K) | $\text{ε(σ)}$ | $\text{ε(S)}$ | $\text{ε(PF)}$ | $\text{ε(}\kappa\text{)}$ | $\varepsilon(ZT)$ |
| --- | --- | --- | --- | --- | --- |
| 300 | 4.21% | 0.55% | 8.71% | 4.09% | 19.25% |
| 373 | 2.83% | 0.46% | 5.95% | 4.48% | 14.89% |
| 473 | 2.52% | 0.53% | 5.47% | 3.36% | 12.83% |
| 523 | 2.25% | 0.34% | 4.70% | 2.41% | 10.56% |
| 573 | 2.54% | 0.53% | 5.50% | 2.65% | 12.22% |
| 623 | 2.97% | 0.67% | 6.52% | 1.74% | 13.49% |
| 673 | 2.77% | 0.55% | 5.95% | 0.82% | 12.01% |
| 723 | 2.56% | 0.50% | 5.51% | 0.86% | 11.15% |
| 773 | 2.68% | 0.42% | 5.62% | 1.63% | 11.71% |

**References**

[1] G. Kresse, D. Joubert, From ultrasoft pseudopotentials to the projector augmented-wave method, *Physical Review B* 59, (1999): 1758-1775. <https://doi.org/10.1103/PhysRevB.59.1758>.

[2] F. Tran, P. Blaha, Accurate Band Gaps of Semiconductors and Insulators with a Semilocal Exchange-Correlation Potential, *Physical Review Letters* 102, (2009): 226401. <https://doi.org/10.1103/PhysRevLett.102.226401>.
